# Mitofusin 1 Drives Preimplantation Development by Enhancing Chromatin Incorporation of Histone H3.3

**DOI:** 10.1002/advs.202414985

**Published:** 2025-03-16

**Authors:** Xiao‐yan Shi, Yu Tian, Yu‐fan Wang, Yi‐ran Zhang, Ying Yin, Qing Tian, Lei Li, Bing‐xin Ma, Ximiao He, Li‐quan Zhou

**Affiliations:** ^1^ Institute of Reproductive Health Tongji Medical College Huazhong University of Science and Technology Wuhan Hubei 430030 China; ^2^ Department of Physiology School of Basic Medicine Tongji Medical College Huazhong University of Science and Technology Wuhan Hubei 430030 China; ^3^ Department of Gynecology and Obstetrics Zhongnan Hospital of Wuhan University Wuhan Hubei 430071 China; ^4^ State Key Laboratory of Stem Cell and Reproductive Biology Institute of Zoology Chinese Academy of Sciences Beijing 100101 P. R. China; ^5^ Reproductive Medicine Center Tongji Hospital, Tongji Medical College Huazhong University of Science and Technology Wuhan Hubei 430030 P. R. China; ^6^ Hubei Key Laboratory of Drug Target Research and Pharmacodynamic Evaluation Huazhong University of Science and Technology Wuhan Hubei 430030 China

**Keywords:** cytoplasmic lattice, H3.3, MFN1, preimplantation, zygotic genome activation

## Abstract

Mitofusin 1 (MFN1) plays a crucial role in mitochondrial fusion and oocyte development. However, its function in preimplantation embryonic development and its potential involvement in epigenetic regulation remain poorly understood. In this study, it is shown that MFN1 interacts with PADI6, a key component of the cytoplasmic lattice in oocytes and early embryos. MFN1 deficiency in mice results in reduced PADI6 levels and decreased expression of translational machinery components, which suppress protein synthesis activity and lower histone H3.3 abundance. These disruptions lead to the failure of male pronucleus formation, aberrant zygotic genome activation, and impaired embryonic development. It is further demonstrated that the MFN1 activator S89 promotes H3.3 incorporation and rescues early development in maternally aged embryos with low MFN1 levels. Additionally, a positive correlation between MFN1 and H3.3 protein levels in early human embryos is observed. Together, these findings provide new insights into MFN1's role in regulating epigenetic reprogramming during preimplantation embryo development.

## Graphical abstract

MFN1 exerts its function partially through histone H3.3 in early embryos. MFN1 colocalizes with PADI6, and its deficiency results in compromised cytoplasmic lattices and ribosomal subunits, leading to declined H3.3 protein level. This, in turn, hinders the incorporation of H3.3 into the embryonic genome, thereby causing failure of male pronucleus formation, 2‐cell arrest, and early embryonic abnormalities.

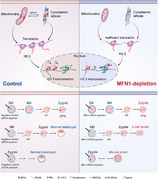

## 1 Introduction

Preimplantation embryonic development begins with the formation of a diploid zygotic genome, created through the fusion of two highly specialized gametes.^[^ ^1^ ^]^ This process triggers extensive epigenomic reprogramming events necessary for the transition to totipotent, mitotic embryos. During this phase, stored maternal factors initiate several key embryonic programs, including maternal RNA degradation, epigenetic reprogramming of histone modifications and variants, zygotic genome activation (ZGA), and the reorganization of subcellular organelles.^[^ ^1a^ ^]^ Gradually, embryonic factors take precedence, driving differentiation, and cell fate decisions.

Some maternal proteins maintain their abundance throughout the preimplantation stages to support early embryonic development.^[^ ^2^ ^]^ For instance, maternal cytoplasmic lattice components assemble into protein complexes that act as reservoirs, facilitating the reorganization of organelles and embryonic programs. This organization is crucial for successful early embryogenesis.^[^ ^3^ ^]^

The histone variant H3.3, encoded by two genes (*H3f3a* and *H3f3b*), produces an identical protein sequence in *Drosophila*, mice, and humans. H3.3 is essential for oocyte growth and early embryonic development. The simultaneous deletion of *H3f3a* and *H3f3b* in growing oocytes causes early primary oocyte stage arrest and cell death.^[^ ^4^ ^]^ Following fertilization, maternal H3.3 rapidly integrates into the paternal genome via HIRA, replacing protamines in the sperm to form the male pronucleus (PN).^[^ ^5^ ^]^ Additionally, H3.3 is critical for ribosomal RNA transcription and DNA replication in the mouse zygote.^[^ ^5d^ ^]^ Knockdown of H3.3 in zygotes leads to morula arrest, a developmental defect that can be rescued by exogenous H3.3 but not by canonical H3.1,^[^ ^6^ ^]^ highlighting the essential and nonredundant roles of H3.3 during embryogenesis. Maternal H3.3 depletion disrupts the expression of ZGA‐related genes and downregulates key pluripotency genes, including *Pou5f1*, in 8–16‐cell embryos.^[^ ^7^ ^]^ Additionally, H3.3 depletion in early embryos compromises genome integrity,^[^ ^4^ ^]^ alters higher order chromatin structure,^[^ ^6^ ^]^ impairs reprogramming activity,^[^ ^8^ ^]^ and disrupts nuclear pore complex assembly.^[^ ^9^ ^]^

The deposition of histone H3.3 is primarily mediated by specific chaperone complexes, including DAXX/ATRX and HIRA/UBN1/CABIN1. These complexes incorporate H3.3 into transcription start sites (TSS), enhancers, telomeres, and centromeres.^[^ ^10^ ^]^ H3.3‐enriched chromatin regions are strongly associated with high transcriptional activity.^[^ ^11^ ^]^ Furthermore, nucleosomes containing H3.3 are less stable than those with canonical histones, enhancing DNA accessibility for transcription machinery.^[^ ^12^ ^]^ Further investigation is needed to identify the upstream regulators and molecular mechanisms governing H3.3 function during distinct stages of early embryonic development.

Mitochondria are dynamic organelles that continuously remodel their architecture through fusion and fission, collectively referred to as mitochondrial dynamics, to adapt to changes in the bioenergetic environment.^[^ ^13^ ^]^ MFN1 and MFN2 are dynamin‐related GTPases essential for mitochondrial fusion in mammalian cells.^[^ ^14^ ^]^ Deletion of *Mfn1* or *Mfn2* results in embryonic lethality during midgestation in mice.^[^ ^15^ ^]^ Notably, depletion of *Mfn1*, but not *Mfn2*, in oocytes leads to follicular depletion and female infertility. This outcome is associated with defective follicle development and impaired oocyte maturation,^[^ ^16^ ^]^ underscoring the critical role of MFN1 in female reproduction. However, the role of MFN1 and its underlying mechanisms in early embryonic development remain unclear. While MFN2 has been shown to regulate metabolism^[^ ^17^ ^]^ and protect cells from death,^[^ ^18^ ^]^ beyond its role in mitochondrial fusion, recent studies have also identified multifunctional roles of mitochondrial fission protein dynamin‐related protein 1 (DRP1). DRP1 acts as a centromere protein F effector to regulate spindle assembly checkpoint activation during mitotic progression.^[^ ^19^ ^]^ These findings suggest that MFN1 may operate through mitochondrial fusion complexes and additional regulatory pathways.

In this study, we demonstrate that MFN1 is continuously expressed during oocyte maturation and preimplantation embryonic development. MFN1 supports early embryo development by maintaining protein synthesis activity, which is essential for histone H3.3 expression. Depletion of MFN1 disrupts histone H3.3 incorporation into the embryonic genome, particularly in open chromatin regions, leading to PN formation failure, ZGA abnormalities, and developmental arrest. Importantly, the activator S89 rescued histone H3.3 incorporation and restored embryonic developmental competence in maternally aged mouse embryos. The multifaceted roles of MFN2 and DRP1 suggest that MFN1 may function through mitochondrial fusion complexes and other regulatory machinery.

## 2 Results

### 2.1 MFN1 Depletion Impairs Oocyte Maturation and Early Embryo Development in Mice

**Figure 1:**
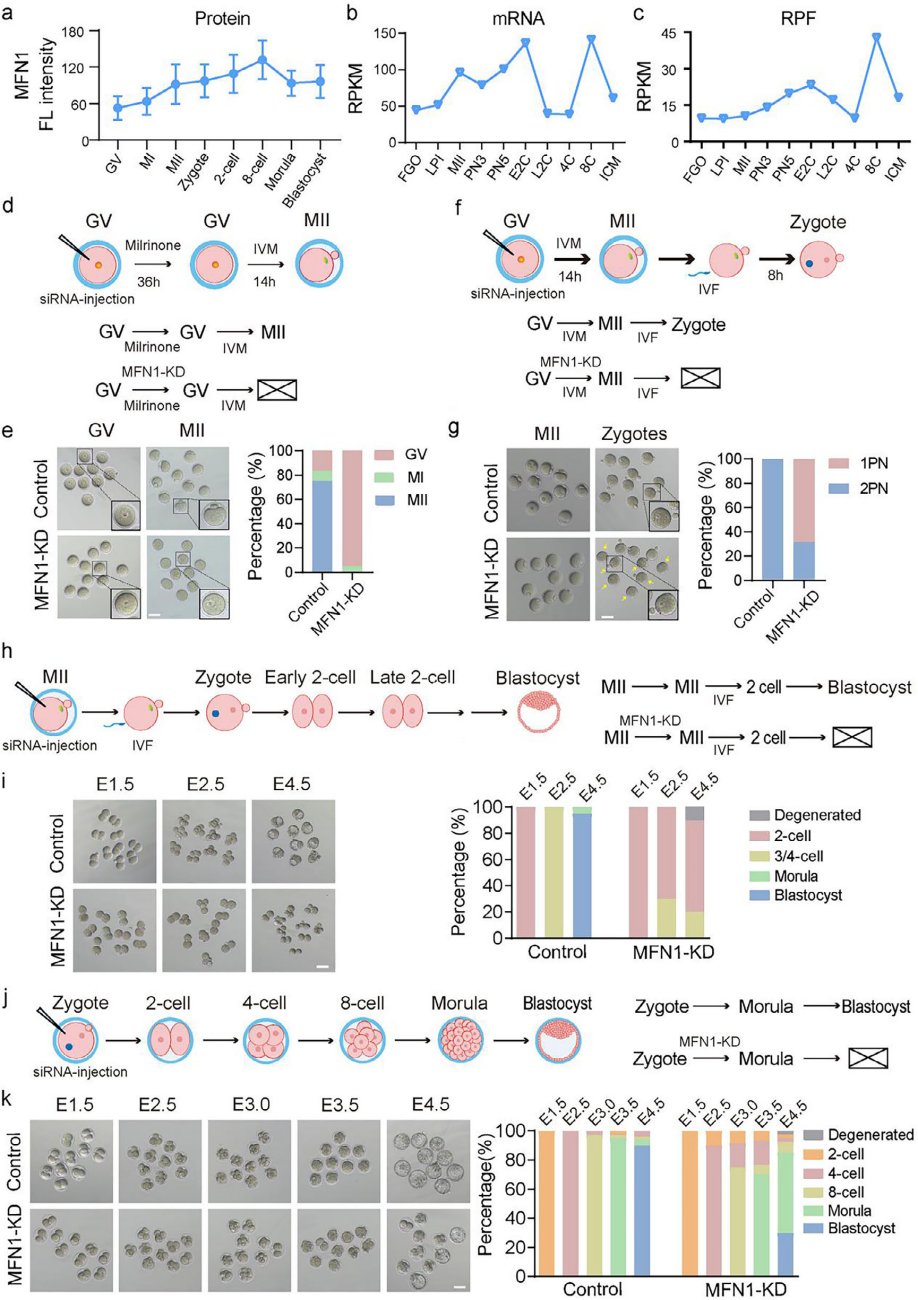
MFN1 is essential for oocytes maturation, fertilization and preimplantation development. a) Quantification of MFN1 immunofluorescence intensity in mouse oocytes and early embryos from Figure S1a of the Supporting Information. Data are presented as mean ± SEM from three independent biological replicates. b,c) The dynamics of *Mfn1* transcript (mRNA‐seq signal, b) and translation (Ribo‐seq signal, RPF, representing the efficiency of translation, c) levels. Data are expressed as the mean of RPKM from two repeats. RPKM, reads per kilobase of bin per million mapped reads; FGO, LPI, and MII, full‐grown, late prometaphase I and MII oocytes; PN3 and PN5, PN3 and PN5 stages of early 1‐cell embryos; E2C and L2C, early and late 2‐cell embryos; 4C and 8C, 4‐ and 8‐cell embryos; ICM, inner cell mass. Public dataset GSE165782 was used for analysis. d) Schematic illustration of microinjection experiment. Mouse GV oocytes were microinjected with negative siRNA or siRNA against *Mfn1*. Following microinjection, oocytes were cultured in medium containing milrinone for 36 h and then transferred in M16 for IVM. e) Representative micrograph of PB1 extrusion (left panel) and stacked bar plots showing fraction of mouse oocytes at the MII stages (right bar graph) in control (*n* = 90) and MFN1‐KD oocytes (*n* = 80). The oocytes from control group successfully extruded PB1, while MFN1‐KD group was mainly arrested at GV stage. The enlarged pictures of one oocyte were shown on the lower right corner. Experiments were repeated at least three times. Scale bar: 80 µm. f) Schematic representation of microinjection experiment. Mouse GV oocytes were microinjected with siRNA against *Mfn1*. After immediate IVM, MII oocytes were fertilized by IVF to examine PN formation. g) Representative images (left panel) and stacked bar plots at the zygotes (right bar graph) of PN formation after fertilization in control (*n* = 95) and MFN1‐KD (*n* = 97) oocytes. Zygotes from control showed normal 2 PN formation, while abnormal 1 PN cells were identified in MFN1‐KD group. Arrows indicate the zygotes with abnormal 1PN. Experiments were repeated at least three times. Scale bar: 80 µm. h) Schematic illustration of microinjection experiment. Mouse MII oocytes were microinjected with siRNA against *Mfn1* and subsequently fertilized by IVF. The developmental potential of MFN1 KD embryos was examined by culture up to the blastocyst stage. i) Representative images (left panel) and rate (right bar graph) of early embryos at the indicated times after fertilization in control (*n* = 82) and MFN1‐KD oocytes (*n* = 79). Most *Mfn1*‐KD embryos failed to develop beyond 2‐cell stage, whereas the control embryos advanced to the blastocyst stage. Experiments were repeated at least three times. Scale bar: 80 µm. j) Schematic illustration of microinjection experiment. Mouse zygotes were microinjected with siRNA against *Mfn1*. k) Representative images (left panel) and rate (right bar graph) of early embryos at the indicated times after fertilization in control (*n* = 120) and MFN1‐KD group (*n* = 125). The majority of the MFN1‐KD embryos reached morula stage but then failed to form blastocysts whereas most of the control embryos developed to blastocyst stage. Experiments were repeated at least three times. Scale bar: 80 µm.

To investigate *Mfn1* expression during mouse oocyte maturation and early embryonic development, we performed immunofluorescence staining to detect MFN1 protein (**Figure** **1**a; Figure S1a, Supporting Information). The results revealed that MFN1 primarily localizes in the cytoplasm of oocytes and blastomeres in early embryos. Its expression gradually increases after fertilization, peaking at the 8‐cell embryo stage. We further analyzed publicly available RNA‐seq and Ribo‐seq datasets,^[^ ^20^ ^]^ which provide insights into mRNA levels and translation activities of individual genes. Consistent with our immunostaining results, *Mfn1* exhibited stable mRNA levels and translation activity across most stages of mouse oocyte maturation and early embryonic development (Figure 1b,c). In contrast, *Mfn2* showed transient expression only during the late 2‐cell to 4‐cell stages, with significantly lower mRNA levels and ribosome‐protected fragment (RPF) counts compared to *Mfn1* (Figure 1b,c; Figure S1b, Supporting Information). These findings highlight the potential regulatory roles of MFN1 in mouse oocytes and early embryos. Interestingly, the localization pattern of MFN1 differed from the distribution of mitochondria (Figure S1a,c, Supporting Information), suggesting that MFN1 may have functions beyond its role in mitochondrial fusion machinery during mouse oocyte maturation and early embryonic development.

To investigate the role of MFN1 during oocyte development, we injected siRNA targeting *Mfn1* into GV oocytes, which were then incubated with milrinone for 36 h (Figure 1d). Both qRT‐PCR and immunoblot analyses confirmed efficient reduction of Mfn1 mRNA and MFN1 protein levels in MFN1 knockdown (KD) oocytes (Figure S1d,e, Supporting Information). Immunostaining further verified that MFN1 signal was significantly diminished in MFN1‐KD oocytes (Figure S1f, Supporting Information). After being inhibited by milrinone for 24 hours, the oocytes were transferred to M16 culture medium without milrinone for in vitro maturation.However, most oocytes remained at the GV stage, and only a small proportion advanced to the MI stage, showing impaired polar body extrusion (PBE) (Figure 1d,e). This result is consistent with previous studies, which reported that targeted deletion of *Mfn1* in female mice causes infertility, attributed to defective folliculogenesis and failure in oocyte maturation.^[^ ^16a,b^ ^]^

To assess MFN1's role postfertilization, we immediately transferred siRNA‐injected GV oocytes to M16 medium and cultured them for 14 h (Figure 1f). The PBE ratio in MFN1‐KD oocytes did not differ significantly from controls (Figure S1g, Supporting Information). Subsequently, the zona pellucida was removed from MII oocytes for in vitro fertilization (IVF). At 8 h postfertilization, 68.33% of zygotes from the MFN1‐KD group displayed a single abnormal pronucleus (1PN), whereas only 31.67% exhibited two pronuclei (2PN) (Figure 1g). This finding confirms that MFN1 is essential for normal PN formation.

To investigate MFN1's role during cleavage‐stage embryo development, we injected super ovulated MII oocytes with siRNA targeting *Mfn1* and subsequently fertilized them via IVF (Figure 1h). Although MFN1‐KD embryos reached the 2‐cell stage, the majority failed to progress beyond this stage, while control embryos advanced to the blastocyst stage (Figure 1i). This observation indicates that MFN1 deficiency causes early embryonic developmental arrest at the 2‐cell stage, suggesting that MFN1 plays a critical role in regulating ZGA.

To further explore MFN1's function during later stages of early embryo development, we performed siRNA‐mediated *Mfn1* knockdown in early zygotes following IVF (Figure 1j). Tracking embryo development revealed that only 30% of MFN1‐KD embryos reached the blastocyst stage, with ≈55% arrested at the morula stage. In contrast, 90% of control embryos progressed to the blastocyst stage (Figure 1k). These results suggest that while MFN1 loss has minimal effect on embryonic development up to the morula stage, it significantly impairs blastocyst formation, indicating that MFN1 likely orchestrates key aspects of early embryonic programming.

In conclusion, our findings establish that MFN1 is essential for oocyte maturation, normal PN formation, and preimplantation development. Future studies will be needed to investigate whether MFN1 regulates early embryo development by orchestrating epigenetic reprogramming events.

### 2.2 MFN1 Depletion in Mouse Oocytes Inhibits Protein Synthesis, H3.3 Deposition, and Male PN Formation

**Figure 2:**
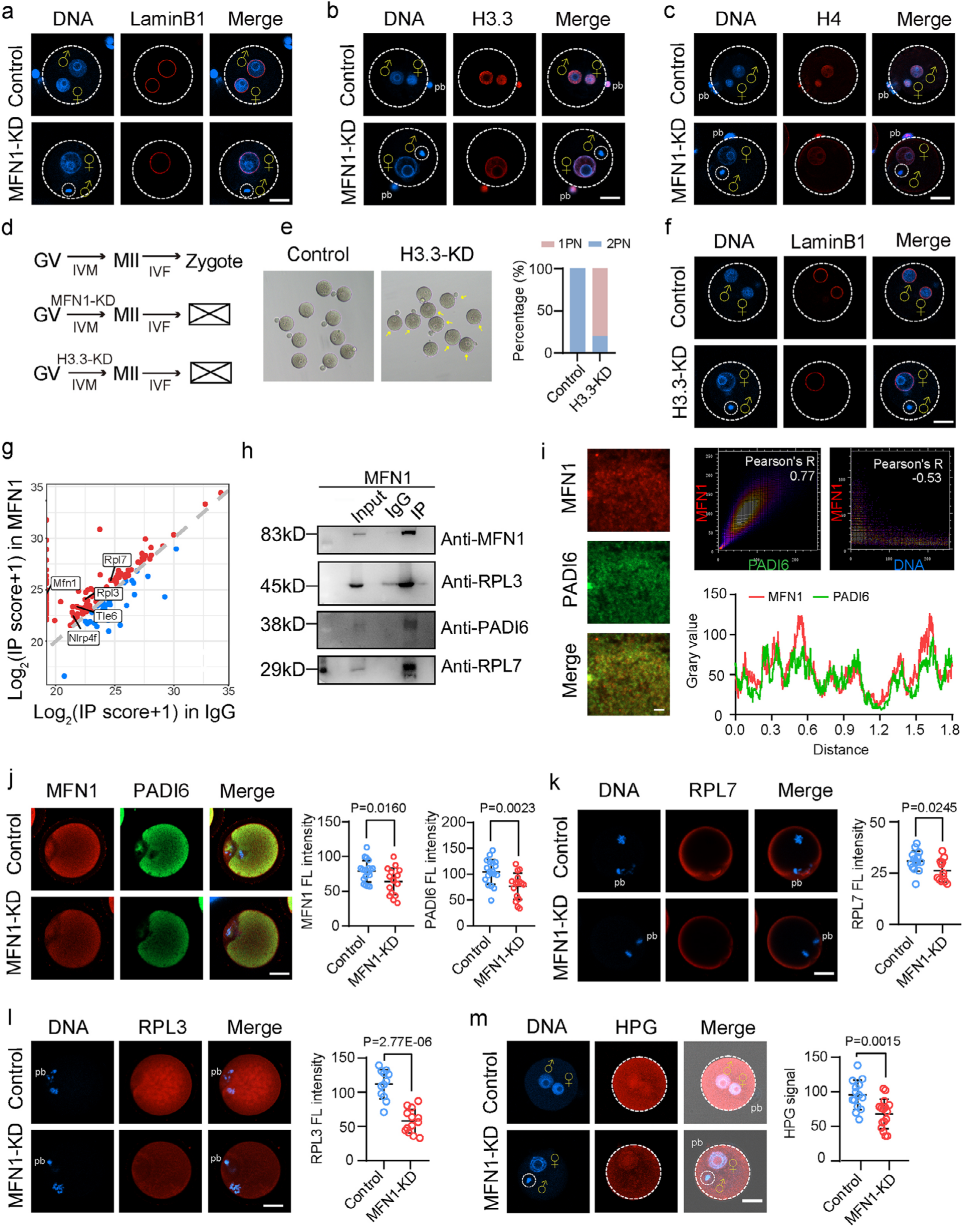
MFN1 supports H3.3 protein abundance and ensures male PN formation. a) Immunostaining of lamin B1 in control and MFN1‐KD mouse zygotes. Scale bar: 25 µm. ♂ represents decondensed sperm head, ♀ represents female PN. b,c) Immunostaining of histone H3.3 (b) and H4 (c) in control and MFN1‐KD mouse zygotes. Scale bar: 25 µm. ♂ represented decondensed sperm head, ♀ represented female PN. d) Schematic representation of microinjection experiment. Mouse GV oocytes were microinjected with siRNA sequences against H3.3 (through targeting both H3.3A and H3.3B). After IVM, MII oocytes were collected for IVF. After 8 h postfertilization, zygotes were collected for further examination. e) Representative images (left panel) and stacked bar plots at the zygotes (right bar graph) of PN formation after fertilization in control (*n* = 103) and H3.3‐KD (*n* = 110) groups. Abnormal 1 PN was identified in MFN1‐KD group while normal 2 PN was observed in control group. Experiments were repeated for at least three times. Arrow indicates the zygotes with abnormal 1PN. Scale bar: 80 µm. f) Immunostaining of lamin B1 in control and H3.3‐KD mouse zygotes. Scale bar: 25 µm. ♂ represents decondensed sperm head, ♀ represents female PN. g) Scatter plot illustration of MFN1‐interacting proteins in mouse MII oocytes identified by IP‐MS with differently enriched proteins highlighted including SCMC components and ribosomal subunits. The proteins with Log_2_ (IP score+1) increased and decreased more than 1 with adjusted *P*‐value less than 0.05 were highlighted in with red and blue red, respectively. h) Co‐immunoprecipitation (Co‐IP) assay showing the interacting proteins of MFN1. i) Representative oocyte expansion images by colabeling using anti‐MFN1 and anti‐PADI6 antibodies and in mouse MII oocytes (left panel). Scale bar: 5 µm. Colocalization quantitative analysis for IF staining of MFN1 with PADI6 (left) and MFN1 with DNA (right) in mouse MII oocytes (right panel). The colocalization quantitative analysis of the MFN1 with DNA serves as a negative control. j) Co‐immunostaining images (above) and quantitative immunofluorescence analyses (below) on MFN1 and PADI6 in mouse MII oocytes from control (*n* = 19) and MFN1‐KD (*n* = 16) groups. Scale bar: 25 µm. k) Representative image of immunofluorescence staining for RPL7 in control and MFN1‐KD MII oocytes (left panel). Quantification signals of RPL7 and RPL3 in control and MFN1‐KD MII oocytes (right panel). Control (*n* = 15), MFN1‐KD (*n* = 12). Scale bar: 25 µm. l) Representative image of immunofluorescence staining for RPL3 in control and MFN1‐KD MII oocytes (left panel). Quantification signals of RPL3 in control and MFN1‐KD MII oocytes (right panel). Control (*n* = 12), MFN1‐KD (*n* = 13). Scale bar: 25 µm. m) Representative immunofluorescence staining images (left panel) and quantification (right panel) of HPG signals in mouse zygotes from control and MFN1‐KD group. Control (*n* = 14), MFN1‐KD (*n* = 16). Scale bar: 25 µm. (j—m) Data are presented as mean ± SEM from at least three times independent experiments. *P*‐values were calculated by two‐tailed Student's *t*‐tests.

MFN1 depletion in MII oocytes resulted in defective 2PN formation (Figure 1g). Immunofluorescence staining for Lamin B1 confirmed the failure of male PN formation in MFN1‐depleted oocytes (**Figure** **2**a). Interestingly, this phenotype mirrors that observed with maternal depletion of histone H3.3, which plays an essential role in oogenesis,^[^ ^4^ ^]^ spermatogenesis,^[^ ^21^ ^]^ and early embryogenesis.^[^ ^22^ ^]^ Previous studies have shown that maternal H3.3 rapidly incorporates into the paternal genome to facilitate male PN formation after fertilization.^[^ ^5^, ^6^ ^]^ The absence of H3.3 in oocytes disrupts male PN formation.^[^ ^5d^ ^]^ Furthermore, H3.3 directly associates with ZGA loci, and its proper expression in early embryos is critical for orchestrating ZGA activity and establishing totipotency.^[^ ^23^ ^]^ These findings raise the intriguing possibility that the failure of male PN formation from MFN1 depletion may be linked to H3.3 insufficiency in early embryos.

To explore this hypothesis, we examined H3.3 incorporation following fertilization in MFN1‐depleted oocytes. As shown in Figure 2b,c, H3.3 and H4 signals were absent from the male genome and were notably diminished in the female PN in MFN1‐depleted zygotes. To determine whether the failure of male PN formation in MFN1‐depleted zygotes resulted from maternal H3.3 loss, we coinjected siRNAs targeting *H3f3a* and *H3f3b* into GV oocytes, followed by oocyte maturation and fertilization (Figure 2d,e). These genes encode the identical H3.3 protein sequence. The H3.3 KD oocytes showed a substantial reduction in H3.3 mRNA levels (Figure S2a, Supporting Information). Notably, the PBE rate was comparable between control and H3.3‐depleted oocytes after 14 h of culture (Figure S2b, Supporting Information). The matured MII oocytes were subsequently used for IVF. As anticipated, the rate of 2PN formation was significantly reduced in late zygotes with H3.3 deficiency (Figure 2e). A similar failure of male PN formation was observed in H3.3‐KD oocytes (Figures 1g and 2e,f). In line with MFN1 depletion, zygotes from the MFN1‐KD group displayed abnormal mitochondrial aggregation and altered mitochondrial membrane potential (MMP) (Figure S2c,d, Supporting Information). These observations promoted us to investigate how MFN1 depletion results in the loss of H3.3 in the sperm genome. To elucidate the molecular mechanisms underlying MFN1 function in zygotes, we performed co‐immunoprecipitation (Co‐IP) using MII oocytes, followed by mass spectrometry to identify components of MFN1‐containing protein complexes. This analysis identified 84 proteins with antibody intensities higher than the IgG control group (Figure 2g), and enrichment analysis revealed that these proteins were primarily involved in ribosomal subunit biogenesis, ribosome assembly, mRNA processing, and stabilization (Figure S2e, Supporting Information). In the potential interacting proteins of MFN1, we noticed the subcortical maternal complex (SCMC) and ribosomal proteins (RPs) (Figure 2g).

To validate these findings, we conducted additional Co‐IP experiments followed by Western blotting (WB). This experiment confirmed that the anti‐MFN1 antibody precipitated not only MFN1 but also PADI6, RPL7, and RPL3 (Figure 2h). Immunofluorescence analysis, combined with cell expansion and colocalization quantitative analysis, revealed that MFN1 colocalized with PADI6 in mouse MII oocytes (Figure 2i). Further investigation showed that both MFN1 and PADI6 signals were significantly reduced in MFN1‐KD oocytes compared to controls (Figure 2j). A previous study reported a reduction in 39 ribosomal subunits in *Padi6* ^−/−^ oocytes.^[^ ^3e^ ^]^ Comparison of these 39 subunits with our mass spectrometry data revealed 29 overlapping ribosomal subunits (Figure S2f, Supporting Information). This finding suggests that MFN1 facilitates the function of the PADI6‐containing cytoplasmic lattice in accumulating translation factors essential for early embryonic development. Consistent with this, immunofluorescence staining showed reduced levels of cytoplasmic lattice components (TLE6, OOEP) and ribosomal proteins (RPL7, RPL3) following MFN1 depletion (Figure 2k,l; Figure S3a,b, Supporting Information). Additionally, MFN1 depletion led to a significant reduction in cortical F‐actin thickness (Figure S3c, Supporting Information). Collectively, our results support the role of MFN1 in maintaining PADI6 and ribosomal protein levels to sustain early embryonic development.

**Figure 3:**
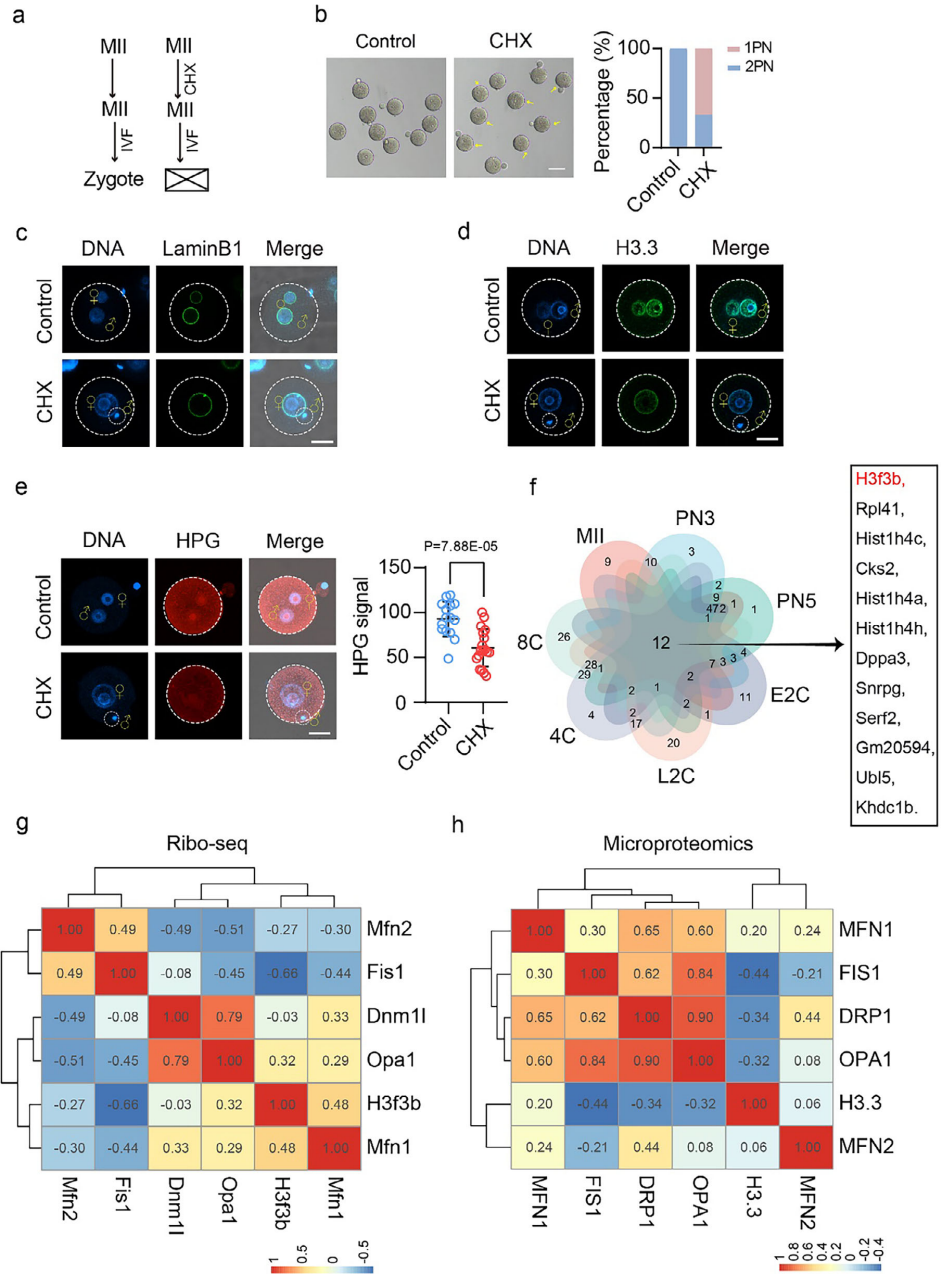
MFN1 contributes to protein synthesis activity in zygotes. a) Schematic of experimental design of fertilized oocytes with or without CHX treatment. MII Oocytes were incubated in M2 medium with DMSO (control) or CHX (CHX treatment) for 6 h before proceeding to IVF. b) Representative images (left panel) and quantification (right panel) of PN formation rates in zygotes with or without CHX treatment. Control (*n* = 98), CHX treatment (*n* = 106). Oocytes treated with CHX failed to form normal 2PN zygotes after fertilization. Arrows indicate the zygotes with abnormal 1PN. Scale bar: 80 µm. c,d) Representative immunostaining images of Lamin B1 (c**o**) and H3.3 (d) in control and CHX treatment zygotes. e) Representative images reveal a substantial decrease in HPG signals in CHX treated (*n* = 18) mouse zygotes compared to control (*n* = 15) zygotes. Above: representative images of HPG signals. Scale bar: 25 µm. Below: quantified HPG signals in mouse zygotes. f) The Venn diagram shows 12 common genes among MII, PN3, PN5, E2C, L2C, 4C, 8C stage in mouse. Public dataset GSE165782 was used for analysis. g) Heatmap of Pearson correlation coefficients for genes related to mitochondria dynamics and *H3f3b* in mouse oocytes and early embryos by public Ribo‐seq dataset GSE165782. h) Heatmap of Pearson correlation coefficients for abundance of proteins related to mitochondria dynamics and H3.3 in human early embryos by public microproteomics dataset PXD042633. (e) Data are presented as mean ± SEM from at least three times independent experiments. *P*‐values were calculated by two‐tailed Student's *t*‐tests.

The decreased levels of ribosomal subunits in MFN1‐deficient oocytes suggest impaired translation activity in zygotes. To investigate this, we performed an l‐homopropargylglycine (HPG) incorporation assay to measure nascent protein synthesis in zygotes. MFN1‐depleted zygotes showed a significant reduction in protein synthesis, as indicated by the HPG signal (Figure 2m), which could contribute to aberrant male PN formation upon MFN1 depletion. To further explore this, we treated MII oocytes with the translation inhibitor cycloheximide (CHX) (**Figure** **3**a,b). We observed that 67.5% of zygotes failed to form a male PN, and these were also devoid of H3.3 following CHX treatment (Figure 3b–d). The reduction in protein synthesis activity after CHX treatment was confirmed in zygotes by the HPG incorporation assay (Figure 2e).

We next investigated how translation activity is linked to H3.3 incorporation into the mouse genome. We analyzed proteome data from early mouse embryos treated with CHX,^[^ ^24^ ^]^ and performed Gene Ontology (GO) analysis on the top 100 translated proteins, ranked by protein fold change after CHX treatment (Figure S3d, Supporting Information). The analysis revealed an overrepresentation of the GO term “protein–DNA complex organization” (Figure S3e, Supporting Information), with *H3f3b* ranked 52nd. Additionally, we examined reported Ribo‐seq data^[^ ^20^ ^]^ for translation activity in genes from the MII stage to the 8‐cell stage. The results showed that H3.3 is continuously expressed, with *H3f3b* expression levels significantly higher than those of *H3f3a* (Figure S3f, Supporting Information). Notably, the translation activity of *H3f3b* ranked within the top 100 genes at various stages, including MII, PN3, PN5, early 2‐cell, late 2‐cell, 4‐cell, and 8‐cell (Figure 3f). In total, 12 overlapping genes were identified among the top 100 genes with the highest translation efficiency across these stages, including *Hist1h4a*, *Hist1h4c*, and *Hist1h4* *h*, which encode histone H4. H3.3 is known to play a critical role in early embryonic development^[^ ^25^ ^]^ and must form dimers with histones H4 to exert its epigenetic functions.^[^ ^26^ ^]^ Furthermore, correlation analysis of the translation activities of genes associated with mitochondrial dynamics and *H3f3b* revealed a positive correlation between *Mfn1* and *H3f3b* (Figure 3g). Similarly, reported microproteomics dataset by mass spectrometry confirmed positive correlation of protein abundance of MFN1 and H3.3 in human early embryos (Figure 3h). These findings suggest that H3.3 protein is maintained by sustained translation throughout early embryo development, is highly sensitive to changes in translation activity, and strongly correlates with MFN1 protein level.

In summary, these results demonstrate that MFN1 depletion impairs protein synthesis, reduces H3.3 levels, and inhibits male PN formation and embryonic development beyond the 1‐cell stage.

### 2.3 MFN1 Deficiency Leads to Aberrant Histone Modifications and ZGA Occurrence in 2‐Cell Mouse Embryos

**Figure 4:**
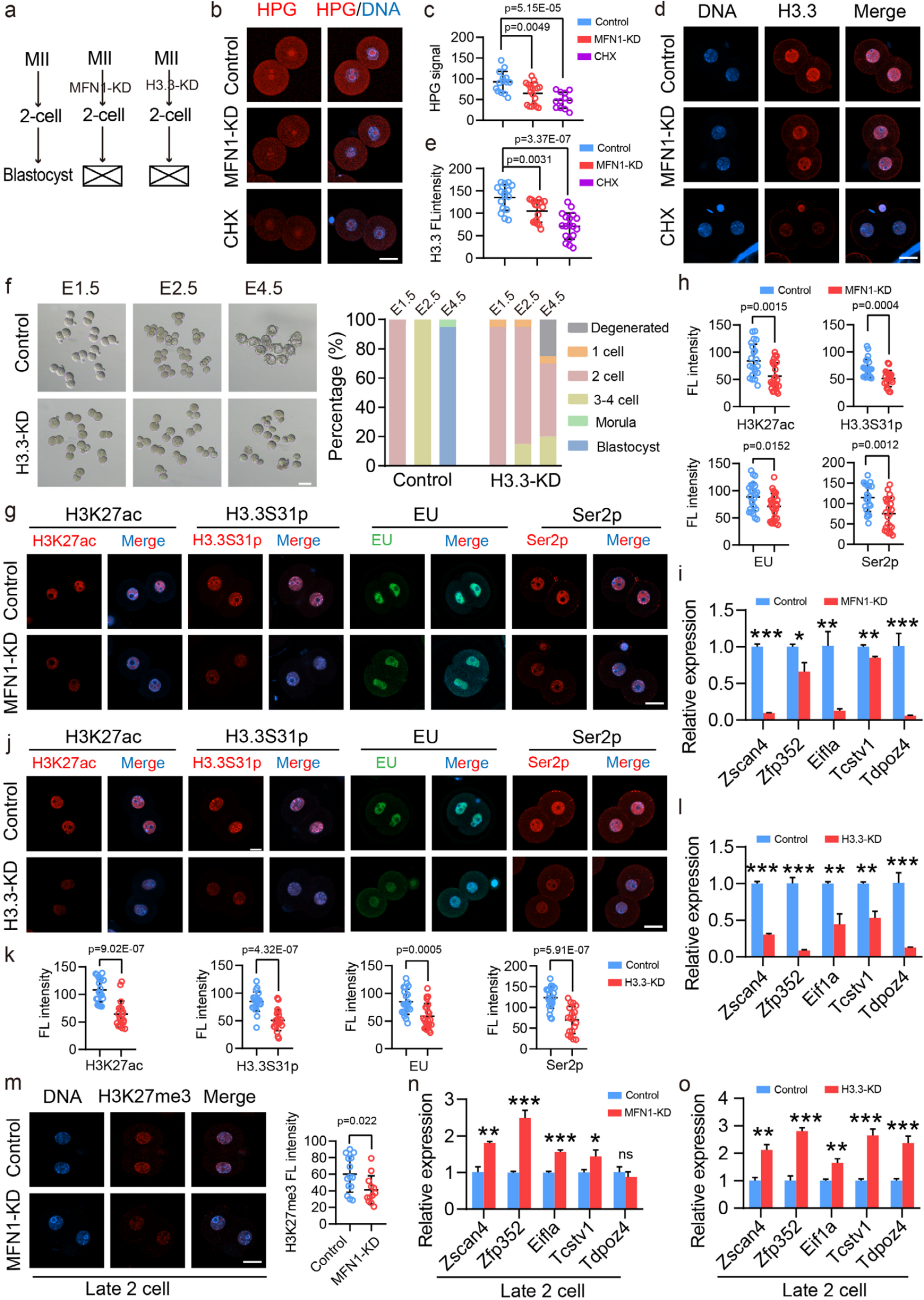
MFN1 facilitates H3.3 expression, and is essential for correct expression of ZGA genes at early and late 2‐cell stages. a Schematic representation of microinjection experiment using siRNA against *Mfn1* or H3.3 (H3.3A and H3.3B). Generally, mouse MII oocytes were microinjected with siRNA and immediately fertilized by IVF using normal sperm. b) Representative images of the HPG signals from control, MFN1‐KD and CHX treated early 2‐cell embryos. Scale bar: 25 µm. c) Fluorescence intensity of the HPG signals from control (*n* = 14), MFN1‐KD (*n* = 18) and CHX treatment (*n* = 12) early 2‐cell embryos. d) Immunofluorescence of H3.3 in control, MFN1‐KD and CHX treated early 2‐cell embryos. Scale bar: 25 µm. e) Quantification of H3.3 in control (*n* = 16), MFN1‐KD (*n* = 16) and CHX treated (*n* = 18) early 2‐cell embryos. f) Representative images (left panel) showing development of early embryos from control (*n* = 97) and H3.3‐KD (*n* = 106) groups at different time points. Scale bar: 80 µm. Quantification (right panel) of the developmental rate at indicated time points from three independent experiments. Most of embryos failed to develop beyond 2‐cell stage after H3.3 depletion, whereas the control embryos advanced to the blastocyst stage. g) Representative images of H3K27ac, H3.3S31p, EU and Ser2p in control and MFN1‐KD early 2‐cell embryos. Scale bar: 25 µm. h) Quantification of H3K27ac, H3.3S31p, EU and Ser2p intensities. Control (*n* = 22, *n* = 24, *n* = 24, *n* = 20), MFN1‐KD (*n* = 24, *n* = 20, *n* = 26, *n* = 22). i) qRT‐PCR results for expression levels of typical ZGA genes in control (*n* = 5) and MFN1‐KD (*n* = 5) early 2‐cell embryos. The expression levels were normalized to *Gapdh*, which served as an internal control. j,k) Representative images (j) and quantification of (k) of H3K27ac, H3.3S31p, and Ser2p in early 2‐cell embryos from control (*n* = 19, *n* = 21, *n* = 21, *n* = 24) and H3.3 KD (*n* = 19, *n* = 20, *n* = 23, *n* = 18) groups. Scale bar: 25 µm. l) qRT‐PCR results for expression levels of typical ZGA genes in control (*n* = 5) and H3.3‐KD (*n* = 5) early 2‐cell embryos. The expression levels were normalized to *Gapdh*, which served as an internal control. m) Representative images (left) and immunofluorescence intensity analysis (right) of H3K27me3 in control (*n* = 14) and MFN1‐KD (*n* = 12) late 2‐cell embryos. Scale bar: 25 µm. n) The relative expression of typical ZGA genes in control (*n* = 5) and MFN1‐KD (*n* = 5) late 2‐cell embryos. The expression levels were normalized to *Gapdh*, which served as an internal control. o) The relative expression of typical ZGA genes in control (*n* = 5) and H3.3‐KD (*n* = 5) late 2‐cell embryos. The expression levels were normalized to *Gapdh*, which served as an internal control. (c,e,h,i,k–o) Data are presented as mean ± SEM from at least three independent experiments. *P*‐values were calculated through two‐tailed Student's *t*‐tests.

Given that fertilization failure in MFN1‐depleted zygotes was linked to reduced H3.3 levels, we sought to determine whether the mechanisms underlying developmental defects in 2‐cell embryos resemble those observed at the zygote stage. MFN1 depletion in cleavage‐stage embryos caused a block at the 2‐cell stage (Figures 1i and 3a). Examination of early 2‐cell embryos revealed that protein synthesis activity, as measured by HPG intensity, and the levels of PADI6 and H3.3 were reduced following MFN1 depletion (**Figure** **4**b–e; Figure S4a, Supporting Information), consistent with our observations at the zygote stage (Figure 2e,j,m).

To assess the impact of protein synthesis activity in cleavage embryos, we treated early 2‐cell embryos with CHX. Nascent protein synthesis was examined by HPG incorporation, and a dramatic decline in protein synthesis activity was observed in CHX‐treated embryos (Figure 4b,c). Furthermore, we noted a reduction in H3.3 intensity in CHX‐treated embryos (Figure 4d,e). Next, we performed knockdown experiments in mouse MII oocytes using siRNA targeting H3.3, followed by IVF (Figure 4f). The results showed a significant decrease in both *Mfn1* and H3.3 levels, at both the mRNA and protein levels, in MFN1‐KD and H3.3‐KD embryos (Figure S4a,b; Figure S4c,d, Supporting Information). Notably, ≈80% of H3.3‐KD embryos was arrested at the 2‐cell stage, whereas control embryos developed to the 3–4‐cell stage. Only a small fraction of H3.3‐KD embryos progressed to the 3–4‐cell stage, even when control embryos reached the blastocyst stage (Figure 4f). This phenotype closely resembled that observed in MFN1‐KD embryos (Figure 1i).

Proper histone modifications are crucial for normal early embryo development. To explore how MFN1 modulates histone modifications in early embryos, we performed immunostaining in early 2‐cell embryos from control and MFN1‐depleted groups. Notably, MFN1 depletion caused a substantial reduction in H3K27ac signal in early 2‐cell embryos (Figure 4g,h). Previous studies have shown that H3K27ac is promoted by phosphorylation of histone H3.3 at serine 31 (H3.3S31p), which in turn enhances transcriptional activity.^[^ ^25^, ^27^ ^]^ Consistent with the decline in H3K27ac, fluorescence staining revealed a noticeable decrease in H3.3S31p intensity in MFN1‐depleted embryos compared to controls (Figure 4g,h). Additionally, we observed reduced intensities of both H3K4me3 and H3K9ac in MFN1‐depleted embryos (Figure S4e,f, Supporting Information). These findings suggest that MFN1 depletion inhibits H3.3S31 phosphorylation, leading to a loss of active histone marks.

Active histone modifications, such as H3K27ac, are associated with transcriptional activation. To assess transcriptional activity, we performed a 5‐ethynyl uridine (EU) incorporation assay, which revealed a significant reduction in the EU signal following MFN1 depletion (Figure 4g,h). Staining for phosphorylated Ser2 of RNA polymerase II (Ser2P), a marker of RNA polymerase II engaged in active transcription, showed significantly reduced levels of actively transcribing RNA polymerase II in the early 2‐cell embryos of the MFN1‐depleted group (Figure 4g,h). Collectively, these results demonstrate that MFN1 depletion impairs transcriptional activation in early 2‐cell embryos.

The abnormal transcriptional activity observed following MFN1 depletion prompted us to investigate whether the expression of ZGA genes was affected. qRT‐PCR results showed that typical ZGA genes, including *Zscan4*, *Zfp352*, *Eif1a*, *Tcstv1*, and *Tdpoz4*, were not fully activated in early 2‐cell embryos lacking MFN1 (Figure 4i).

To determine whether the developmental defects observed in MFN1‐depleted early 2‐cell embryos were due to a reduction in H3.3 protein levels, we conducted similar experiments using H3.3‐KD early 2‐cell embryos. We observed a dramatic decrease in the intensities of H3K27ac, H3.3S31p, H3K4me3, and H3K9ac in H3.3‐KD embryos (Figure 4j,k; Figure S4g,h, Supporting Information). In addition, EU incorporation and Ser2P signals were significantly reduced in these embryos (Figure 4j,k). Furthermore, the expression of typical ZGA genes was also compromised in H3.3‐KD embryos (Figure 4l). These findings suggest that MFN1 depletion inhibits H3.3 protein expression by reducing protein synthesis, leading to decreased active histone marks and impaired ZGA activity in early 2‐cell embryos.

Additionally, we observed that MFN1 deficiency caused a reduction in H3K27me3 intensity (Figure 4m) and an upregulation of typical ZGA gene expression (Figure 4n) at the late 2‐cell stage, a time when ZGA is typically silenced, and H3K27me3 marks genes for transcriptional repression. Similarly, we identified decreased H3K27me3 levels (Figure S4i, Supporting Information) and increased expression of typical ZGA genes (Figure 4o) in H3.3‐KD late 2‐cell embryos. These results suggest that in late 2‐cell embryos, MFN1 facilitates H3.3 expression, which in turn establishes H3K27me3 modification, enabling transcriptional silencing and ZGA shutdown.

In the absence of MFN1, 2‐cell embryos from the MFN1‐KD group exhibited abnormal mitochondrial distribution and membrane potential (Figure S5a,b, Supporting Information). However, KD of H3.3 did not significantly affect mitochondrial distribution or MMP (Figure S5c,d, Supporting Information). Taken together, these results demonstrate that in 2‐cell embryos, MFN1 promotes protein synthesis and H3.3 protein accumulation, which establishes active histone marks to stimulate ZGA at the early 2‐cell stage and repressive histone marks to silence ZGA at the late 2‐cell stage.

### 2.4 MFN1 Facilitates H3.3 Expression and Embryonic Programming in 8‐Cell Mouse Embryos

**Figure 5:**
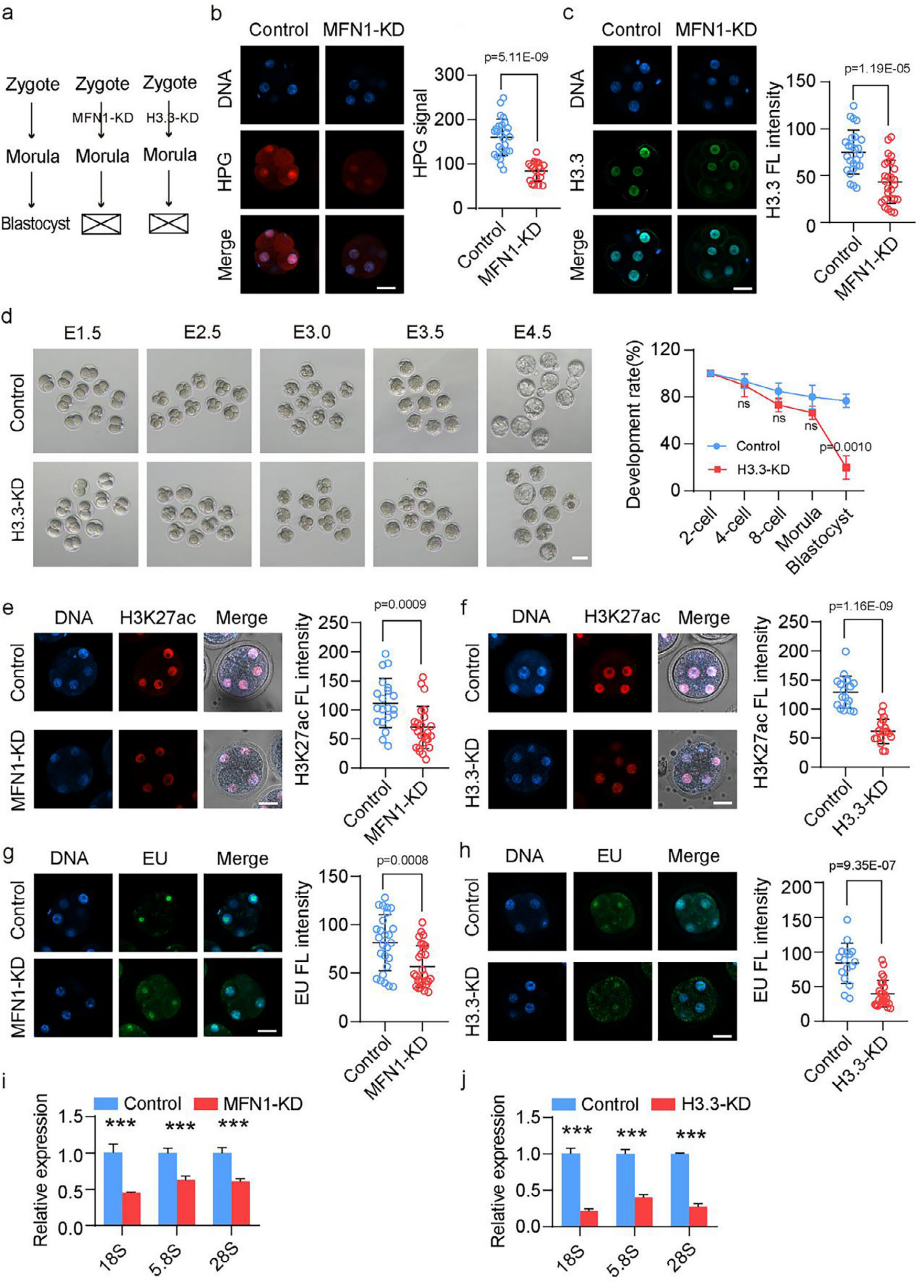
Knockdown of MFN1 in zygote leads to reduced H3.3 protein level at the 8‐cell stage and caused morula arrest. a) Experimental scheme of microinjection experiment in zygotes. Generally, early mouse zygotes were microinjected with siRNA against *Mfn1* or H3.3, followed by in vitro culture until blastocyst stage. b) Immunofluorescence (left panel) and quantification (right panel) of HPG signals in MFN1‐KD (*n* = 19) and control (*n* = 26) 8‐cell embryos. Scale bar: 25 µm. c) Immunofluorescence (left panel) and quantification (right panel) of H3.3 in MFN1‐KD (*n* = 26) and control (*n* = 25) 8‐cell embryos. Scale bar: 25 µm. d) Representative images of early embryonic development of control and H3.3‐KD embryos (left panel). The percentage of embryos that reached the 2‐cell, 4‐cell, 8‐cell, morula, and blastocyst stages at E1.5, E2.5, E3.0, E3.5, and E4.5 was calculated and presented as the developmental rate (right panel). Control, *n* = 114. H3.3‐KD, *n* = 118. Most of embryos from control group developed to blastocyst stage, while H3.3‐KD embryos were mainly arrested at morula stage. e) Representative images (left) and quantification of H3K27ac signals (right) in control (*n* = 20) and MFN1‐KD (*n* = 26) 8‐cell embryos. Scale bar: 25 µm. f) Representative images (left) and quantification of H3K27ac signals (right) in control (*n* = 16) and H3.3‐KD (*n* = 25) 8‐cell embryos. Scale bar: 25 µm. g) Representative images (left) and quantification of EU‐positive nuclear signals (right) in control (*n* = 26) and MFN1‐KD (*n* = 28) 8‐cell embryos. Scale bar: 25 µm. h) Representative images (left) and quantification of EU‐positive nuclear signals (right) in control (*n* = 18) and H3.3‐KD (*n* = 18) 8‐cell embryos. Scale bar: 25 µm. i) qRT‐PCR results showing the RNA levels of 18S, 5.8S, and 28S in 8‐cell embryos after microinjection of siRNA against *Mfn1* in zygotes. *Gapdh* served as the internal control. *n* = 5 embryos in each sample. j) qRT‐PCR results showing the RNA levels of 18S, 5.8S, and 28S in 8‐cell embryo after microinjection of siRNA against H3.3 in zygotes. *Gapdh* served as the internal control. *n* = 5 embryos in each sample. (b–j) Data are presented as mean ± SEM from at least three independent experiments. *P*‐values were calculated by two‐tailed Student's *t*‐tests.

Depletion of MFN1 from the zygote stage led to developmental arrest at the morula stage (Figures 1k and 5a). We then sought to determine whether the underlying mechanisms were consistent with those observed in MFN1‐deficient zygotes and 2‐cell embryos. Given that the MFN1 protein level peaks at the 8‐cell stage during early embryo development (Figure S1a, Supporting Information), we selected this stage for subsequent analysis following *Mfn1* knockdown at the zygote stage.

qRT‐PCR and immunofluorescence staining showed a significant reduction in both mRNA and protein levels of *Mfn1* at the 8‐cell stage after siRNA‐mediated knockdown of *Mfn1* in zygotes (Figure S6a,b, Supporting Information). We then examined protein synthesis activity and H3.3 protein levels in MFN1‐depleted 8‐cell embryos. Both the HPG signal and nuclear H3.3 levels were significantly decreased in these embryos (Figure 5b,c). Furthermore, we assessed the levels of PADI6, other components of the SCMC, and RPs. Immunofluorescence analysis revealed consistent reductions in the signals of MFN1 and PADI6 (Figure S6c, Supporting Information). Similarly, OOEP, TLE6, and RPL7 showed decreased signals in MFN1‐depleted 8‐cell embryos (Figure S6d–f, Supporting Information). These findings align with the observations in MFN1‐depleted zygotes and 2‐cell embryos, further supporting the role of MFN1 in facilitating H3.3 expression and driving the developmental program during the mid‐preimplantation stage. To determine whether reduced H3.3 protein levels contribute to developmental abnormalities at the 8‐cell stage, we injected siRNA targeting H3.3 into early mouse zygotes (Figure 5a,d). qRT‐PCR and immunofluorescence staining revealed a significant decrease in both H3.3 mRNA and protein levels in 8‐cell embryos (Figure S7a,b, Supporting Information). Despite this, we observed that H3.3 depletion had minimal impact on embryo compaction, but significantly reduced the blastocyst developmental rate (**Figure** **5**d), a phenotype consistent with that observed in MFN1‐depleted embryos (Figure 1k). These results suggest that MFN1 depletion weakens H3.3 protein levels, which are crucial for blastocyst formation.

Next, we examined the effects of MFN1 and H3.3 depletion on histone modifications and RNA synthesis. H3K27ac signals were reduced in 8‐cell embryos following the depletion of either MFN1 or H3.3 (Figure 5e,f), indicating that both proteins contribute to chromatin opening in early embryos. To assess nascent RNA synthesis, we performed EU staining at the 8‐cell stage after MFN1 or H3.3 depletion. We observed a marked reduction in EU signals, demonstrating a significant impairment in transcriptional activation (Figure 5g,h). Interestingly, qRT‐PCR analysis of rRNA expression revealed downregulation of 18S, 5.8S, and 28S rRNA in both MFN1‐KD and H3.3‐KD embryos (Figure 5i,j). Additionally, we detected a global decrease in H3K4me3 and H3K36me3 signals in both MFN1‐KD and H3.3‐KD embryos (Figure S7c–f, Supporting Information). In summary, these findings indicate that both MFN1 and H3.3 are essential for establishing active histone marks and RNA synthesis in 8‐cell embryos.

As anticipated, 8‐cell embryos in the MFN1‐KD group exhibited abnormal mitochondrial aggregation, a decrease in mitochondrial DNA (mtDNA) copy number, and disrupted MMP (Figure S8a–c, Supporting Information). Similarly, depletion of H3.3 also resulted in abnormal mitochondrial distribution, reduced mtDNA copy number, and impaired MMP (Figure S8d–f, Supporting Information), highlighting the significant role of H3.3 in early embryonic development.

In summary, these findings suggest that MFN1 facilitates H3.3 expression in 8‐cell embryos, promoting chromatin opening and RNA synthesis.

### 2.5 MFN1 Facilitates H3.3‐Activated Gene Expression in 8‐Cell Mouse Embryos

**Figure 6:**
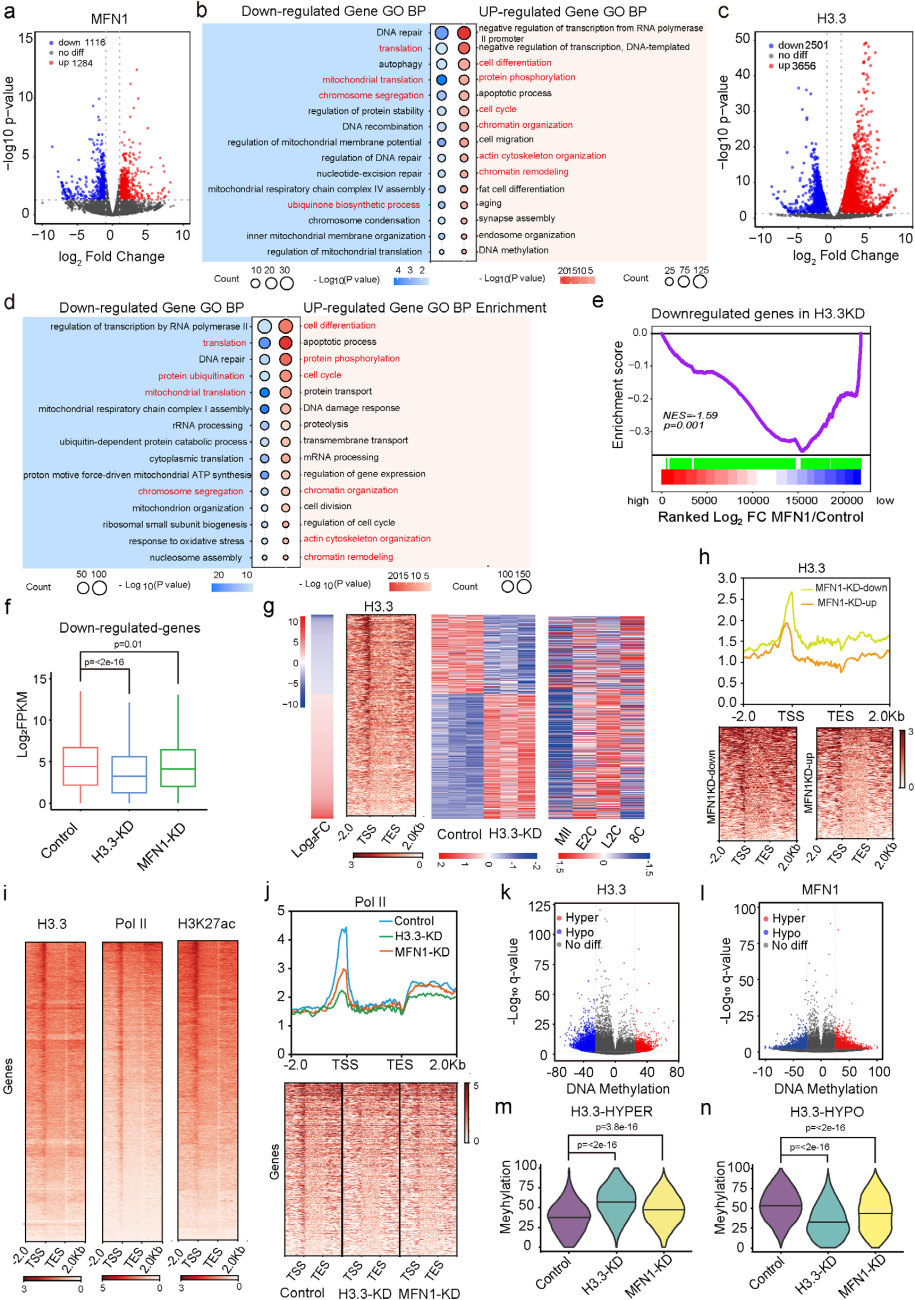
MFN1 facilitates the deposition of H3.3 at transcriptionally active genes. a) Volcano plot showing transcriptome changes in MFN1‐KD 8‐cell embryos. Red and blue dots indicate upregulated and downregulated genes upon *Mfn1* knockdown, respectively. b) GO analysis of upregulated and downregulated genes in MFN1‐KD 8‐cell embryos for biological process (BP) enrichment. The sizes of the circles represent the number of genes. Red indicated the same enriched pathways with H3.3‐KD 8‐cell embryos. c) Volcano plot showing transcriptome changes in H3.3‐KD 8‐cell embryos. Red and blue dots indicate upregulated and downregulated genes upon H3.3 knockdown, respectively. d) GO analysis of upregulated and downregulated genes in H3.3‐KD 8‐cell embryos for BP enrichment. The sizes of the circles represent the number of genes. Red indicated the same enriched pathways with MFN1‐KD 8‐cell embryos. e) GSEA analysis of enrichment of genes repressed by H3.3 deficiency at 8‐cell stage in DEGs of MFN1‐KD embryos. Red, downregulated genes. Blue, upregulated genes. NES: normalized enrichment scores, The Kolmogorov–Smirnov statistic was used for calculation of *P*‐value. f) Boxes plot of mRNA levels by RNA‐seq depicting downregulated genes in H3.3‐KD 8‐cell embryos were also significantly repressed in MFN1‐KD 8‐cell embryos. Mann–Whitney *U* test was used to calculate *P*‐values. g) Heatmap of dysregulated genes upon H3.3 knockdown at 8‐cell stage, ranked by Log_2_Foldchange values. H3.3 occupancy at corresponding genic loci in 8‐cell embryos, gene expression in control and H3.3‐KD 8‐cell embryos, and average gene expression levels during development from MII oocytes to early embryos by RNA‐seq (public dataset GSE165782) were also shown by heatmap. FC, fold change. h) Density plot of H3.3 occupancy at 2 kb downstream of TSS to 2 kb upstream of TES of downregulated/upregulated genes by *Mfn1* knockdown in 8‐cell embryos (upper panel), and corresponding heatmap (lower panel). i) Heatmap of H3.3, RNA polymerase II, and H3K27ac enrichment at 2 kb upstream/downstream of all genes from the TSS to the TES in 8‐cell embryos, ranked by H3.3 enrichment. j) Density plot of RNA polymerase II and H3K27ac enrichment at 2 kb upstream/downstream of all genes from the TSS to the TES in control, H3.3‐KD and MFN1‐KD 8‐cell embryos (upper panel), and corresponding heatmap (lower panel). k) Scatter plot showing hyper‐DMRs and hypo‐DMRs in MFN1‐KD 8‐cell embryos, indicated by red and blue dots, respectively. l) Scatter plot showing hyper‐DMRs and hypo‐DMRs in H3.3‐KD 8‐cell embryos, indicated by red and blue dots, respectively. m) Violin plot showing significant increase of DNA methylation at hyper‐DMRs by H3.3 knockdown in both H3.3‐KD and MFN1‐KD 8‐cell embryos. Mann–Whitney *U* test was used to calculate *p*‐values. n) Violin plot showing significant decrease of DNA methylation at hypo‐DMRs by H3.3 knockdown in both H3.3‐KD and MFN1‐KD 8‐cell embryos. Mann–Whitney *U* test was used to calculate *P*‐values.

To further elucidate the molecular mechanisms underlying MFN1's function during early embryonic development and its interaction with H3.3, we performed transcriptome analysis using low‐input RNA sequencing on 8‐cell embryos from control, MFN1‐KD, and H3.3‐KD groups. Strong correlations were observed across the three biological replicates for each group (Figure S9a,b, Supporting Information). Overall, we identified 1284 upregulated and 1116 downregulated genes in the MFN1‐KD group (**Figure** **6**a,b; Figure S9c,d, Supporting Information), and 3656 upregulated and 2501 downregulated genes in the H3.3‐KD group (Figure 6c,d; Figure S9e,f, Supporting Information). As expected, *Mfn1* and *H3f3a*/*H3f3b* were among the most downregulated genes in the MFN1‐KD and H3.3‐KD groups, respectively (Figure S9d,f, Supporting Information), confirming the efficiency of gene knockdown at the 8‐cell stage.

To further investigate the functions of differentially expressed genes (DEGs), we performed GO analysis. This analysis revealed that the upregulated genes following MFN1 depletion were enriched in pathways related to the negative regulation of transcription from the RNA polymerase II promoter, transcription regulation, chromatin organization, cell differentiation, protein phosphorylation, chromatin remodeling, the cell cycle, and aging. In contrast, downregulated genes were primarily involved in mitochondrial translation, chromosome segregation, assembly of mitochondrial respiratory chain complex IV, DNA recombination, regulation of protein stability, and translation (Figure 6b). GO analysis of H3.3 target genes revealed upregulation in biological processes such as cell differentiation, protein phosphorylation, cell cycle progression, protein transport, proteolysis, chromatin remodeling, gene expression regulation, and chromatin organization. Conversely, downregulated genes were enriched in pathways related to RNA polymerase II‐mediated transcription regulation, translation, protein ubiquitination, mitochondrial translation, mitochondrial respiratory chain complex I assembly, cytoplasmic translation, chromosome segregation, and ribosomal small subunit biogenesis (Figure 6d). Interestingly, the analysis of sequencing results from MFN1‐depleted embryos showed similar changes in GO pathways compared to H3.3 knockdown, including cell differentiation, chromatin remodeling, chromatin organization, mitochondrial respiratory chain complex assembly, and chromosome segregation (Figure 4b,d). These findings suggest that MFN1 plays a crucial role in the proper expression of H3.3‐dependent genes.

After depletion of MFN1, downregulated genes were enriched in cellular components (CCs) and molecular functions (MFs) primarily associated with organelles, GTPase binding, catalytic activity, and lyase activity (Figure S10a, Supporting Information). These findings suggest dysfunction of organelles and a deficiency in protein degradation. Upregulated genes were enriched in CC and MF linked to the cytosol, nucleoplasm, cytoplasm, nucleus, and transcription‐related activities, such as transcription coactivators, regulatory factors, DNA binding, and RNA polymerase II‐dependent DNA binding (Figure S10b, Supporting Information). This pattern indicates impaired gene expression and transcription. Kyoto Encyclopedia of Genes and Genomes (KEGG) analysis of MFN1 target genes revealed that downregulated genes were involved in protein processing in the endoplasmic reticulum, and the biosynthesis of cofactors and amino acids. In contrast, upregulated genes were associated with pathways related to MAPK signaling, Foxo signaling, Hippo signaling, and Wnt signaling (Figure S10c,d, Supporting Information), suggesting compromised protein biosynthesis, cell cycle regulation, cell proliferation, and embryonic development.

Upon depletion of H3.3, downregulated genes showed enrichment in CC and MF linked to organelles, protein binding, and transferase activity (Figure S11a, Supporting Information), further implying organelle dysfunction and a deficiency in protein degradation. Upregulated genes were enriched in CC and MF related to organelles, nucleoplasm, cytosol, nucleus, and transcription‐related functions, such as DNA binding, nucleotide binding, RNA polymerase II, and DNA binding transcription factor activity (Figure S11b, Supporting Information). These results suggest defects in organelle function, gene expression, and transcription.

KEGG analysis of H3.3 target genes identified downregulated terms related to the ribosome, oxidative phosphorylation, ubiquitin‐mediated proteolysis, and motor proteins. Upregulated terms were linked to various diseases, as well as MAPK signaling and Wnt signaling (Figure S11c,d, Supporting Information), indicating defects in protein synthesis and degradation, mitochondrial function, and embryonic polarity. Additionally, we observed that genes involved in cell fate determination, such as *Otx2*, were decreased in both MFN1‐ and H3.3‐depleted 8‐cell embryos.

GSEA analysis of the enrichment of downregulated genes following H3.3 knockdown revealed a significant overlap between H3.3‐activated genes in 8‐cell embryos and MFN1‐activated genes in the same developmental stage (Figure 6e). Furthermore, a box plot demonstrated that genes downregulated by H3.3 knockdown were also downregulated in the MFN1‐KD group (Figure 6f). Similarly, genes upregulated by H3.3 knockdown exhibited upregulation in the MFN1‐KD group (Figure S11e, Supporting Information).

These transcriptome findings indicate that MFN1 deficiency disrupts the expression of embryonic genes at the 8‐cell stage, likely through H3.3‐mediated transcriptional regulation.

### 2.6 MFN1 Facilitates H3.3 Deposition at Actively Transcribed Genic Loci in 8‐Cell Mouse Embryos

To examine the impact of MFN1 deficiency on epigenetic modifications, we performed ChIP‐seq experiments on MFN1‐KD and H3.3‐KD 8‐cell embryos. By integrating RNA‐seq data from H3.3‐KD embryos with ChIP‐seq data for H3.3 occupancy at the 8‐cell stage, we observed that the promoter regions of genes downregulated in H3.3‐KD embryos displayed significantly higher enrichment of H3.3 compared to upregulated genes (Figure 6g). This finding suggests that H3.3 is closely associated with transcriptional activation at the 8‐cell stage. RNA‐seq analysis of gene expression during normal development, from MII oocytes to 8‐cell embryos, further supported this conclusion. Genes downregulated by H3.3 knockdown were normally upregulated between the late 2‐cell stage and the 8‐cell stage (Figure 6g), confirming that H3.3 deficiency impairs transcriptional activation during this critical period. Conversely, genes upregulated by H3.3 knockdown were typically downregulated from the late 2‐cell stage to the 8‐cell stage (Figure 6g). This reduced expression during normal development is likely driven by newly synthesized genes at the 8‐cell stage, which promote transcript degradation and developmental progression.

Density plots showed a reduction in H3.3 occupancy at genic loci of H3.3‐KD DEGs in MFN1‐depleted embryos (Figure S11f, Supporting Information). Similarly, we observed decreased H3.3 occupancy at the loci of H3.3‐activated genes in MFN1‐depleted embryos (Figure S11g, Supporting Information). Notably, after MFN1 depletion, downregulated genes exhibited higher H3.3 binding than upregulated genes (Figure 6h). These findings highlight the crucial role of H3.3 in MFN1‐mediated gene activation. Our results demonstrate that MFN1 promotes H3.3 occupancy at chromatin in 8‐cell embryos, enabling transcriptional activation of target genes.

Next, we found that genic regions with higher H3.3 occupancy also displayed increased RNA polymerase II and H3K27ac enrichment in 8‐cell embryos (Figure 6i). This observation supports the notion that H3.3 binds to actively transcribed genes to facilitate transcriptional activation at this stage. To further investigate how MFN1 and H3.3 affect transcriptional activity in early embryos, we analyzed the distribution of RNA polymerase II and H3K27ac in MFN1‐KD and H3.3‐KD 8‐cell embryos. Consistent with our earlier findings, RNA polymerase II and H3K27ac occupancy at genic regions decreased upon MFN1 or H3.3 depletion (Figure 6j; Figure S11h, Supporting Information), suggesting impaired enrichment of these factors at gene promoters required for transcriptional activation in 8‐cell embryos.

We then examined whether DNA methylation patterns were altered in MFN1‐ and H3.3‐depleted 8‐cell embryos. Whole‐genome bisulfite sequencing (WGBS) analysis revealed 12 259 hypomethylated differentially methylated regions (hypo‐DMRs) and 3142 hypermethylated DMRs (hyper‐DMRs) in H3.3‐KD embryos. In MFN1‐KD embryos, 7071 hypo‐DMRs and 9584 hyper‐DMRs were identified (Figure 6k,l). Notably, the DNA methylation levels of hyper‐DMRs and hypo‐DMRs in H3.3‐KD embryos were similarly elevated or reduced, respectively, in the MFN1‐KD group (Figure 6m,n). These results indicate that MFN1 deficiency induces corresponding changes in DNA methylation patterns similar to those observed in H3.3‐deficient embryos at the 8‐cell stage.

Above results demonstrate that MFN1 promotes the incorporation of H3.3 into embryonic chromatin, coordinating epigenetic modifications and transcriptional activity in 8‐cell embryos.

Interestingly, if the reduction of H3.3 caused by MFN1 depletion leads to fertilization failure and early embryonic developmental defects, then introducing exogenous H3.3 into MFN1‐depleted oocytes may reverse some of phenotypic abnormalities. To test this hypothesis, we coinjected H3.3 cRNA and siRNA targeting *Mfn1* into oocytes or zygotes, and then assessed 2PN formation and blastocyst rates in the resulting embryos (H3.3‐rescue group). Indeed, we observed significant improvement in 2PN zygote formation (Figure S12a,b, Supporting Information) and blastocyst formation (Figure S12c–e, Supporting Information) following coinjection of H3.3 cRNA, indicating that exogenous H3.3 partially rescued the developmental defects induced by MFN1 depletion.

### 2.7 MFN1 Activator Elevates H3.3 Protein Levels and Improves Early Development of Maternally Aged Mouse Embryos

**Figure 7:**
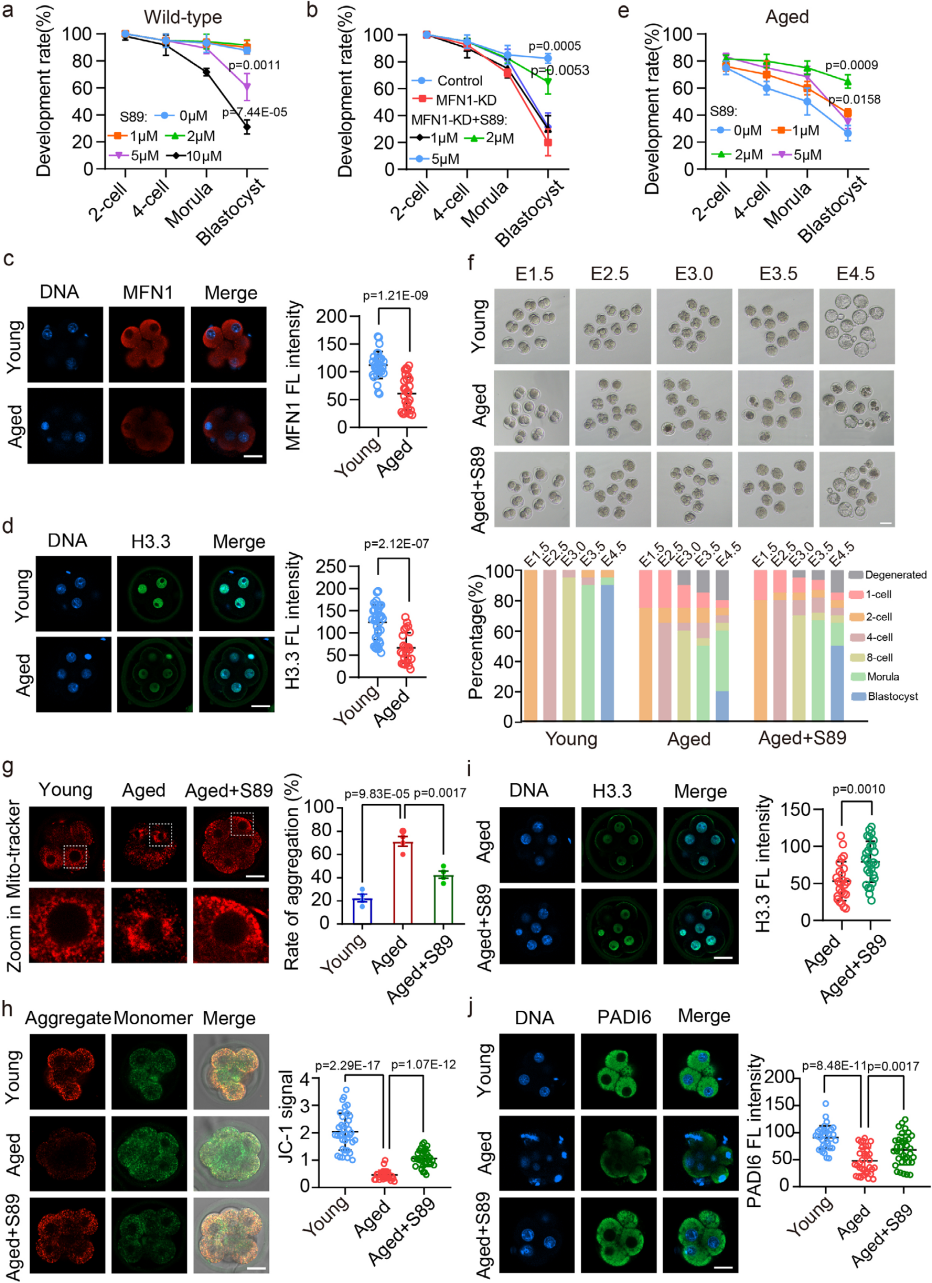
S89 promotes early development of MFN1‐KD embryos and maternally aged embryos. a) The line chart showing the percentages of embryos that reached the 2‐cell, 4‐cell, morula, and blastocyst stages at E1.5, E2.5, E3.5, and E4.5 under different concentrations of S89 (0, 1, 2, 5, and 10 µm) in wild‐type mice. 0 µm, *n* = 80. 1 µm, *n* = 80. 2 µm, *n* = 90. 5 µm, *n* = 90, and 10 µm, *n* = 85. b) The line chart showing the percentage of embryos that reached the 2‐cell, 4‐cell, morula, and blastocyst stages at the indicated time points in the control group, MFN1‐KD group, and S89‐rescue group with different concentrations (0, 1, 2, and 5 µm). Control, *n* = 90. MFN1‐KD, *n* = 88. 0 µm, *n* = 93. 1 µm, *n* = 86. 2 µm, *n* = 95. 5 µm, *n* = 87. c) Immunostaining (left panel) and fluorescence intensities (right panel) of MFN1 in 8‐cell embryos derived from young (*n* = 36) and aged female mice (*n* = 24). Scale bar: 25 µm. d) Immunostaining (left panel) and fluorescence intensities (right panel) of H3.3 in 8‐cell embryos from young (*n* = 30) and aged female mice (*n* = 28). Scale bar: 25 µm. e) Quantifications of embryos reaching the 2‐cell, 4‐cell, morula, and blastocyst stages at E1.5, E2.5, E3.5, and E4.5, respectively. These percentages were then used to represent the developmental rates of embryos in maternally aged mice treated with different concentrations of S89 (0, 1, 2, and 5 µm). 0 µm, *n* = 63. 1 µm, *n* = 65. 2 µm, *n* = 72. 5 µm, *n* = 69. f) Representative images (left panel) and quantification (right panel) showing the development of maternally young embryos (*n* = 100), maternally aged embryos (*n* = 90), and maternally aged embryos with S89 supplementation (*n* = 104) at indicated time points. Scale bar: 80 µm. g) Mitochondrial distribution (left panel) and aggregation ratio (right panel) in 8‐cell embryos from young (*n* = 52), aged (*n* = 47) and S89 supplemented aged (*n* = 60) group. Scale bar: 25 µm. h) JC1 staining (left panel) in young (*n* = 39), aged (*n* = 26), and S89 supplemented aged (*n* = 33) 8‐cell embryos to detect MMP. MMP level is indicated by red/green ratio (right panel). Scale bar: 25 µm. i) Immunofluorescence staining (left panel) and fluorescence intensities (right panel) of H3.3 in 8‐cell embryos from aged (*n* = 24) and S89 supplemented aged (*n* = 28) group. Scale bar: 25 µm. j) Immunofluorescence staining (left panel) and fluorescence intensities (right panel) of PADI6 in 8‐cell embryos from young (*n* = 33), aged (*n* = 34), and S89 supplemented aged (*n* = 35) group. Scale bar: 25 µm. (a–j) Data are presented as mean ± SEM from at least three independent experiments. *P*‐value was calculated using two‐tailed Student's *t*‐test.

S89 is a small molecule known to repair mitochondrial dysfunction by specifically activating MFN1 to promote mitochondrial fusion.^[^ ^28^ ^]^ To investigate the effects of S89 on early development in MFN1‐KD embryos, we first assessed the optimal concentration for S89 supplementation in embryo culture medium, based on its effective working concentration reported in the previous study.^[^ ^28^ ^]^ As shown in **Figure** **7**a and S12f (Supporting Information), no significant differences in early embryo development were observed when concentration of 0, 1, or 2 µm was used. However, at 5 µm, S89 did not alter the proportion of morula but significantly impaired blastocyst formation. At the concentration of 10 µm, S89 markedly reduced the proportion of morula (Figure 7a; Figure S12f, Supporting Information). Next, we investigated whether S89 could rescue early embryonic development in MFN1‐KD embryos (Figure 7b; Figure S12g, Supporting Information). Supplementation with 2 µm S89 significantly improved the blastocyst formation rate in the MFN1‐KD group (Figure 7b; Figures S12g and S13a, Supporting Information). Analysis of mitochondrial distribution and MMP in 8‐cell embryos revealed that S89 supplementation enhanced mitochondrial activity in MFN1‐KD embryos (Figure S13b,c, Supporting Information). To further evaluate the effects of S89, we stained 8‐cell embryos from control, MFN1‐KD, and MFN1‐KD with S89 supplementation groups for H3.3 and PADI6. Fluorescence imaging and signal quantification demonstrated that S89 supplementation increased H3.3 and PADI6 levels in MFN1‐KD embryos (Figure S13d,e, Supporting Information). These findings indicate that S89 partially restored mitochondrial function and histone H3.3 expression disrupted by MFN1 depletion, thereby promoting early embryonic development.

Recent studies have shown that chromosomal histone H3.3 levels decline with maternal age.^[^ ^26^ ^]^ Consistent with this, our analysis of 8‐cell embryos from aged female mice revealed reduced H3.3 and MFN1 intensities compared to embryos from young females (Figure 7c,d). This prompted us to test whether S89 supplementation could improve the developmental potential of maternally aged embryos. We collected zygotes from young and aged female mice using IVF, cultured the embryos with or without S89, and recorded their developmental stages at the indicated times. Our results showed that early embryos from aged female mice exhibited significantly lower developmental potential than those from young females. Importantly, compared with supplementation with 1 and 5 µm S89, supplementation with 2 µm S89 significantly restored the developmental progression of embryos from aged females (Figure 7e,f; Figure S13f, Supporting Information). To investigate mitochondrial function at E3.0, we analyzed mitochondrial distribution and activity in embryos. In the young group, mitochondria were evenly distributed throughout the cytoplasm and concentrated near the nucleus. In contrast, embryos from the aged group exhibited mitochondrial clustering in the cytoplasm, with a significantly higher proportion of abnormal distribution compared to the young group. Notably, S89 supplementation partially restored normal mitochondrial distribution in the aged group (Figure 7g). We also assessed MMP at E3.0. Consistent with the mitochondrial distribution patterns, S89 supplementation partially recovered MMP levels in aged embryos (Figure 7h). Together, these findings demonstrate that S89 supplementation improves mitochondrial function in maternally aged 8‐cell embryos by restoring mitochondrial distribution and MMP levels, thereby enhancing early embryonic developmental capacity.

Next, we investigated whether the improvement in mitochondrial function by S89 supplementation influenced the levels of histone H3.3 and PADI6 in 8‐cell embryos from aged female mice. Fluorescence imaging and quantification at E3.0 revealed that S89 supplementation effectively restored H3.3 and PADI6 levels in maternally aged embryos (Figure 7i,j).

To determine whether the observed mitochondrial dysfunction was a cause or consequence of MFN1 depletion, we examined oocytes subjected to postovulatory aging and H_2_O_2_ treatment, which partially mimic the mitochondrial integrity defects associated with maternal aging. As expected, both the two groups showed impaired mitochondrial distribution and reduced MMP (Figure S14a,b, Supporting Information). However, the protein levels of MFN1 remained unchanged in both postovulatory aged oocytes and H_2_O_2_‐treated oocytes (Figure S14c–e, Supporting Information). These results suggest that mitochondrial dysfunction is the consequence, rather than the cause, of MFN1 depletion.

Our findings demonstrate that maternal aging reduces MFN1 and H3.3 levels, while the MFN1 activator S89 enhances H3.3 protein levels and promotes the development of early embryos from aged female mice.

### 2.8 MFN1 Expression Is Positively Correlated with H3.3 Expression in Early Human Embryos and Is Reduced by Maternal Aging

**Figure 8:**
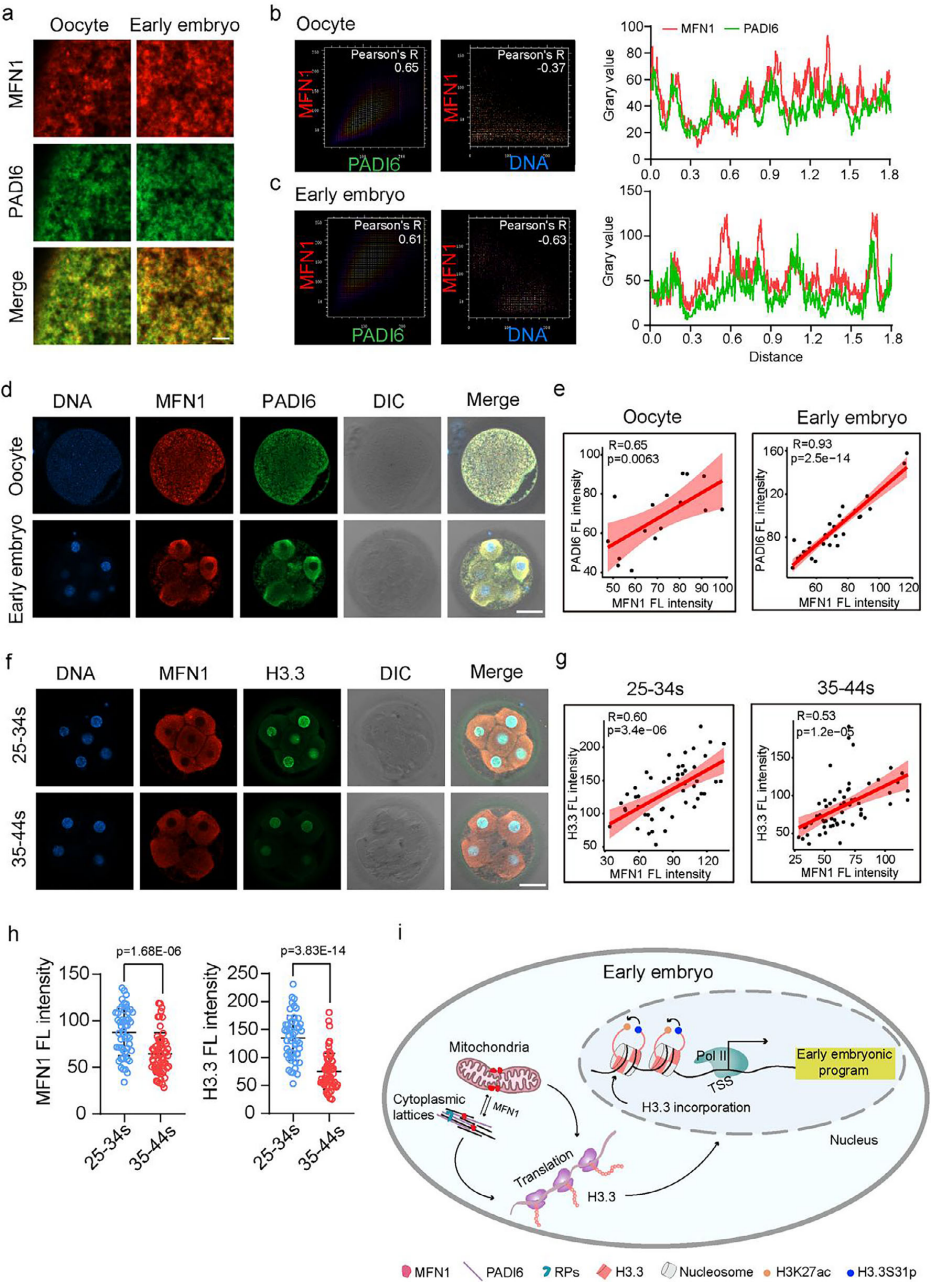
PADI6 and H3.3 protein levels are positively correlated with MFN1 protein level in human early embryos. a) Representative cell expansion images of colabeled MFN1 and PADI6 in human MII oocytes and 4–8 cell embryos. Scale bar: 4 µm. b,c) Colocalization quantitative analysis for IF staining of MFN1 with PADI6 and MFN1 with DNA in human MII oocytes (b) and early embryos (c). The colocalization quantitative analysis of the MFN1 with DNA serves as a negative control. d) Representative co‐immunostaining images of MFN1 and PADI6 in human MII oocytes and 4–8 cell embryos. Scale bar: 40 µm. e) Correlation analysis of PADI6 fluorescence intensity with MFN1 fluorescence intensity in MII oocytes and 4–8‐cell embryos in (b). Red shaded areas represent the 95% confidence intervals. f) Human 4–8‐cell embryos were divided into “25–34 years (25–34s)” and “35–44 years (35–44s)” groups by maternal ages for co‐immunostaining of MFN1 and H3.3. Scale bar: 40 µm. g) The fluorescence intensities of MFN1 and H3.3 signals in “25–34s” (*n* = 51) and “35–44s” (*n* = 60) groups for comparison. Data are presented as mean ± SEM. *P*‐values were calculated by two‐tailed Student's *t*‐tests. h) Correlation analysis of H3.3 fluorescence intensity with MFN1 fluorescence intensity in 4–8 cell embryos from “25–34s” and “35–44s” groups. Red shaded areas represent the 95% confidence intervals. i) Schematic diagram of the mechanism that MFN1 supports H3.3 enrichment at active chromatin regions and regulates preimplantation embryo development. MFN1 interacted with PADI6 and RPs to maintain the global translation level of early embryos, strengthened H3.3 protein level and its incorporation at genic regions, elevated H3.3S31p and H3K27ac to stimulate transcription activity, thereby ensuring the proper early embryonic program.

To investigate MFN1 protein expression in human oocytes and early embryos, we collected clinically discarded human MII oocytes and 4–8‐cell embryos for immunostaining analysis. Immunofluorescence combined with cell expansion and quantitative colocalization analysis revealed that MFN1 colocalized with PADI6 in human MII oocytes and early embryos (**Figure** **8**a–c). Furthermore, immunostaining showed a positive correlation between MFN1 and PADI6 protein levels in both human oocytes and early embryos (Figure 8d,e). Next, we categorized human 4–8‐cell embryos into two maternal age groups: 25–34 years and 35–44 years. We performed co‐immunofluorescence staining for MFN1 and H3.3 in embryos from both groups (Figure 8f). Quantitative analysis revealed a significant decline in MFN1 and H3.3 protein levels in embryos from the “35–44 years” group compared to the “25–34 years” group (Figure 8g). Correlation analysis further confirmed a positive relationship between MFN1 and H3.3 fluorescence intensities in both age groups (Figure 8h).

In summary, our results indicate that MFN1 colocalizes with PADI6 and positively correlates with H3.3 protein levels in human early embryos. Maternal aging reduces MFN1 expression, which may contribute to compromised embryonic development.

## 3 Discussion

In this study, we identified a novel role of mitochondrial dynamics in regulating embryonic development via epigenetic pathways. Our findings demonstrated that the mitochondrial fusion regulator MFN1 supports histone variant H3.3 by maintaining global translational activity in embryos. This process facilitates the deposition of highly translated H3.3 at gene promoter regions, ensuring transcription activation and driving preimplantation embryonic development. Specifically, MFN1 depletion disrupted cytoplasmic lattice components, significantly reduced global translation levels particularly of H3.3 protein and led to PN formation failure, defective ZGA, and aberrant embryonic developmental programs (Figure 8i). Importantly, the adverse effects of MFN1 deficiency were partially rescued by either exogenous H3.3 or supplementation with the MFN1 activator S89, underscoring the critical role of MFN1 in supporting the developmental competence of preimplantation embryos. Collectively, our study highlights the pivotal role of MFN1 in facilitating PN formation, ZGA, and embryonic development through H3.3 expression and enrichment.

While current knowledge of MFN1's role and mechanisms in oocyte and early embryo development remains limited, our findings align with previous studies reporting abnormal oogenesis and mid‐gestational embryonic lethality in *Mfn1* knockout mice.^[^ ^15^, ^16^ ^]^ Consistent with these observations, we found that MFN1 depletion caused arrest at the GV stage, impairing normal meiotic progression. However, the precise mechanisms and broader functions of MFN1 in early embryogenesis warrant further investigation.

To investigate the role of MFN1 in preimplantation embryo development, we first examined its function during fertilization. We observed that MFN1 depletion reduced H3.3 expression, resulting in male PN formation failure after fertilization. Recent studies have shown that the cytoplasmic lattice, which contains PADI6 and SCMC components, serves as a storage site for maternal proteins accumulated during oogenesis and is essential for embryonic development.^[^ ^3e^ ^]^ Our findings indicated that while MFN1 and mitochondria did not fully colocalize, MFN1 partially colocalized with PADI6 in the cytoplasm of oocytes. Disruption of PADI6 or SCMC leads to the loss of the cytoplasmic lattice, resulting in reproductive disorders and complete female infertility in both mice and humans.^[^ ^29^ ^]^ In line with this, we found that MFN1 depletion decreased protein translation activity by impairing PADI6 function and ribosomal subunit assembly.

Previous studies have highlighted the role of cytoplasmic lattices in the oocyte‐to‐embryo transition (MZT) in both mice and humans.^[^ ^2^, ^30^ ^]^ However, the molecular mechanisms governing the dynamic assembly and function of cytoplasmic lattices affected by MFN1 depletion remain unclear. Our findings may provide a valuable framework for further exploring these mechanisms. Additionally, H3.3 protein levels are highly sensitive to changes in protein translation activity.^[^ ^24^ ^]^ Notably, chromatinization of the sperm genome occurs before DNA replication in the early zygote, and H3.3 acting as a maternal factor is the only histone H3 variant available to replace protamines and integrate into the chromatin prior to the DNA synthesis phase via a replication‐independent pathway.^[^ ^31^ ^]^ Consistent with these findings, our results demonstrate that the reduction in protein translation following MFN1 depletion led to a significant decrease in H3.3 expression, thereby impairing male PN formation. This suggests that MFN1 plays a critical role in stabilizing protein translation and facilitating H3.3 incorporation into chromatin.

Previous research on mitochondrial fusion proteins primarily focused on mitochondrial activity and subcellular organelle defects.^[^ ^32^ ^]^ A key finding of this study is that MFN1 depletion significantly reduces H3.3 expression, a histone variant crucial for ZGA and the embryonic developmental program.^[^ ^7^, ^33^ ^]^ We observed diminished H3.3 deposition in early 2‐cell embryos following *Mfn1* knockdown, accompanied by decreased H3.3S31p and H3K27ac modifications, which led to abnormal ZGA gene expression. H3.3 modulates transcription through mechanisms such as facilitating H3K27ac at regulatory elements via H3.3S31p, thereby promoting transcriptional activation.^[^ ^27^ ^]^ Conversely, stable gene silencing is associated with chromatin compaction and repressive histone modifications, such as H3K27me3, which inhibit transcriptional machinery and regulate gene imprinting in a DNA methylation‐independent manner.^[^ ^34^ ^]^ Our findings revealed that MFN1 depletion in late 2‐cell embryos reduced H3.3 levels and H3K27me3 modifications, impairing the silencing of ZGA genes. Similar effects on ZGA genes were observed in 2‐cell embryos following H3.3 depletion, suggesting that MFN1 orchestrates ZGA gene regulation by modulating H3.3 expression.

Furthermore, *Mfn1* knockdown in mouse zygotes led to reduced H3.3 deposition, disrupted the embryonic program, and resulted in morula arrest. These findings align with previous studies, which reported that diminished H3.3 deposition in zygotes causes chromosomal overcondensation, missegregation, and failure to progress to the blastocyst stage during preimplantation development.^[^ ^5^, ^6^ ^]^ Given the parallels between MFN1 and H3.3 depletion, we propose that MFN1 exerts its role in mid‐preimplantation development primarily through its regulation of H3.3.

To investigate the relationship between MFN1 and H3.3, we performed low‐input RNA sequencing and ChIP‐seq analyses to examine their roles in regulating the embryonic program in 8‐cell embryos. H3.3 primarily localized to regions of active genes, with a strong enrichment at promoter regions.^[^ ^35^ ^]^ Our analysis revealed that, after H3.3 depletion, H3.3 preferentially bound to the promoters of downregulated genes. Similarly, genes downregulated by H3.3 knockdown exhibited repressed expression in MFN1‐depleted embryos. Notably, the downregulated genes following MFN1 depletion displayed increased H3.3 binding. These findings suggest that H3.3 occupancy plays a crucial role in transcription activation mediated by both MFN1 and H3.3 during the 8‐cell stage.

Previous studies have shown that histone H3.3 is incorporated into chromatin at enhancers and promoters, correlating with active histone marks such as H3K27ac, H3K9ac, H3K4me3, H3K4me1, and H2A.Z.^[^ ^5^, ^36^ ^]^ Our results align with these findings, demonstrating that H3.3 is positively correlated with transcriptionally active chromatin marks, including H3K27ac and RNA polymerase II. Interestingly, embryos from both the H3.3‐ and MFN1‐depleted groups exhibited reduced levels of RNA polymerase II and H3K27ac. This suggests that gene expression in 8‐cell embryos is altered under these conditions. Our study further shows that MFN1 depletion leads to a decrease in H3.3 deposition, resulting in reduced transcriptional activity in early embryos. This decline is accompanied by diminished enrichment of RNA polymerase II and H3K27ac at the promoters of downregulated genes, ultimately causing developmental arrest. Importantly, exogenous H3.3 supplementation partially rescued the defects in male PN formation and early embryo development caused by MFN1 depletion.

Ovarian function and female fertility decline significantly with age, particularly between the ages of 35 and 39, due to a reduction in ovarian reserve and oocyte quality.^[^ ^37^ ^]^ Females with MFN1‐deficient oocytes experience anovulation, as folliculogenesis arrests at the secondary‐to‐antral follicle stage. This phenotype worsens with age, leading to accelerated follicular depletion.^[^ ^16^ ^]^ Mitochondrial dysfunction is a hallmark of age‐related oocyte quality decline, contributing to pregnancy loss and developmental disorders in offspring.^[^ ^38^ ^]^ In line with these findings, we observed reduced MFN1 levels in early embryos of maternally aged mice, oocytes, and early embryos from middle‐aged women. Supplementation with the MFN1 activator S89 reversed mitochondrial dysfunction, restored H3.3 and PADI6 protein levels, and corrected developmental defects in early embryos caused by maternal aging‐associated MFN1 insufficiency. Notably, our results indicated that an excessive amount of S89 might not necessarily be beneficial for female reproduction. Our results suggest that MFN1 may serve as a valuable therapeutic target for alleviating fertility disorders and age‐related declines in female reproduction.

In conclusion, our study demonstrates that MFN1 functions, in part, through histone H3.3 during early embryonic development in mice. Supplementation with the MFN1 activator S89 enhances H3.3 expression and improves the developmental competence of preimplantation embryos from maternally aged mice. These findings provide new insights into the interaction between mitochondrial fusion regulators and epigenetic modifications, offering potential strategies for improving embryonic development and female fertility in the context of maternal aging.

## 4 Experimental Section

### Mouse Breeding

Young (6‐to‐8‐week‐old) and naturally aged (12‐to‐13‐month‐old) female ICR mice, and 10‐to‐20‐week‐old male ICR mice were purchased from SiPeiFu (Beijing, China). All the animal procedures were approved by the Institutional Animal Care and Use Committee of Tongji Medical College, Huazhong University of Science and Technology (IACUC No. 4096). All animals were housed in the specific pathogen‐free facility with unrestricted access to food and water, with a temperature of 22–25 °C, 50–70% humidity, and a 12 h light/12 h dark cycle at the animal center of Tongji Medical College, Huazhong University of Science and Technology. All experiments with mice were conducted according to the Guide for the Care and Use of Laboratory Animal guidelines.

### Mouse Oocyte Collection and Culture

For the superovulation assay, female ICR mice were intraperitoneally primed with 10 IU of pregnant mare serum gonadotropin (PMSG), After 48 h, ovaries were mechanically cut with ophthalmic scissors in M2 medium (Sigma‐Aldrich, USA). Then the intact germinal vesicle (GV) oocytes, which were free of cumulus cells, were individually transferred to M16 medium (Sigma‐Aldrich, USA) with or without 2.5 µm Milrinone overlaying with mineral oil and cultured at 37 °C with 5% CO_2_.

For H_2_O_2_ treatment, 100 µm H_2_O_2_ was chosen based on previous study.^[^ ^39^ ^]^ GV oocytes cultured with H_2_O_2_ and 2.5 µm milrinone for 30 min were collected for immunofluorescence staining or Western blot examination.

For postovulatory aging experiments, GV oocytes were cultured with 2.5 µm milrinone for 24 or 48 h before being collected for immunofluorescence staining or Western blot examination.

### In Vitro Fertilization (IVF) and Early Embryo Culture

Superovulation was performed in female ICR mice by injection of 10 IU of PMSG, followed by 10 IU of human chorionic gonadotropin (hCG) 48 h later. After 16 h, the cumulus–oocyte complex (COC) was collected from the ampulla of oviducts from the superovulated mice, and transferred to the HTF medium at 37 °C with 5% CO_2_. Sperm were released from the dissected cauda epididymis of male ICR mice before insemination and capacitated for 1 h in HTF (Nanjing Aibei Biotechnology, China) at 37 °C with 5% CO_2_. Subsequently, the capacitated sperm were added into COCs drops in HTF at 37 °C with 5% CO_2_. Then fertilized oocytes were collected and cultured in KSOM medium (Nanjing Aibei Biotechnology, China). The early 2‐cell, later 2‐cell, 4‐cell, 8‐cell, morula, and blastocyst stages embryos were obtained on E1.5, E2.0, E2.5, E3.0, E3.5, and E4.5 post hCG injection, respectively. For the detection of translational effects on fertilization, early embryos were cultured in KSOM medium containing 20 µg mL^−1^ cycloheximide (CHX, Selleck, China) for 6 h.

### H3.3 cDNA Construct and In Vitro Transcription (IVT)

Endogenous mouse H3.3 protein is encoded by two genes, *H3f3a* and *H3f3b*, and these genes produce an identical protein. H3.3 gene fragments were amplified in vitro via PCR using pVAX1 plasmid expressing flag‐tagged Drosophila H3.3 cDNA as the template for IVT. Subsequently, capped cRNA of H3.3 was synthesized using the mMESSAGE mMACHNE T7 Ultra kit (Thermo Fisher Scientific, USA) and purified by precipitation with LiCl, followed by dissolution in nuclease‐free water and storage at −80 °C. pVAX1 IVT primer sequences are displayed in Table S1 of the Supporting Information.

### Cytoplasmic Microinjection

Oocytes (GV/MII) or zygotes were incubated in the M2 medium covered with mineral oil. Then, ≈5–10 pl of 25 µm siRNA against specific genes (Genepharma, China) was injected into the cytoplasm of oocytes or zygotes. The nontargeting siRNA was injected as the control counterpart.

After injection, GV oocytes were incubated for 36 h in 2.5 µm milrinone to allow the degradation of mRNA by siRNA and then transferred to milrinone‐free M16 medium to resume meiosis for subsequent experiments. Microinjected MII oocytes were transferred to M2 medium for a brief 30 min recovery and then cultured shortly in acidic Tyrode's Solution (pH 2.5) to eliminate zona pellucida before IVF. The microinjected zygotes were transferred into KSOM for incubation at 37 °C under 5% CO_2_. For coinjection experiment, 500 ng µL^−1^ H3.3 cRNA was injected with siRNA against *Mfn1* into oocytes or zygotes to test the impact of exogenous H3.3 on development. siRNA sequences against *Mfn1*, *H3f3a*, *H3f3b*, and negative control siRNA are provided in Table S2 of the Supporting Information.

### S89 Treatment of Early Embryos

To ascertain the safe concentration of S89 on the development of early embryo, the blastocyst rate of early embryo development with S89 treatment was examined at different concentrations (0, 1, 2, 5, and 10 µm) based on previous study on S89 usage.^[^ ^28^ ^]^

To test the protective effect of S89, the zygotes following siRNA injection and those from maternally aged mice were cultured in KSOM supplemented with S89 at different concentrations (0, 1, 2, and 5 µm).

### Collection of Human Oocyte and Early Embryos

Human MII oocytes and early embryos were obtained from Reproductive Medicine Center, Tongji Medical College, Huazhong University of Science and Technology. A total of 26 oocytes from oocyte donation and 35 frozen‐thawed 4–8‐cell embryos abandoned by patients were included in this study. Ethical approval for the study was obtained by the CEIC (Ethics Committee for Clinical Research) of Reproductive Medicine Center, Tongji Medical College, Huazhong University of Science and Technology (No. S207). All people included in the study gave informed consent.

### Co‐Immunoprecipitation (Co‐IP) and Mass Spectrometry (MS)

For co‐IP, 20 ovaries from juvenile mice at postnatal day 7 to 14 (P7‐P14) or 800 oocytes were homogenized in chilled IP lysis buffer (Beyotime, China) with protease inhibitor cocktail (Beyotime, China) and centrifuged at 12 000 × *g* for 20 min. The supernatants were collected and incubated with relevant antibodies (anti‐MFN1 antibody and IgG) overnight at 4 ℃ with gentle rotation. The precleared protein A/G magnetic beads (Vazyme, China) were supplemented and cocultured for 30 min. The supernatants were then gently removed and the beads were washed with lysis buffer. The beads containing samples were subjected for MS analysis, or eluted with the elution buffer and then boiled with 5× loading buffer for Western blot analysis.

### Cell Expansion Assay

Cell‐expansion immunofluorescence was performed to examine colocalization of proteins. MII oocytes were incubated in acidic Tyrode's solution at 37 °C for 30 s to remove zona pellucidae and transferred to fresh M2 medium for 10 min. MII oocytes were then equilibrated in hypotonic buffer (30 mm Tris (pH 8.2), 50 mm sucrose, 0.5 mm phenylmethylsulphonyl fluoride, 5 mm EDTA, 0.5 mm DTT, and 17 mm trisodium citrate dihydrate) for 30 min. Then the MII oocytes were incubated with 2% paraformaldehyde (PFA) and 0.15% Triton X‐100 in a humidified chamber for 60 min at room temperature and washed with 0.4% Photo‐Flo (Kodak, Japan) for subsequent immunostaining.

### Immunofluorescence Staining and Confocal Microscopy

For immunofluorescence staining, oocytes/embryos were fixed with 4% PFA for 30 min, permeabilized with 0.5% Triton X‐100 for 20 min and blocked with 1% bovine serum albumin for 1 h. Subsequently, samples were incubated with primary antibodies at 4 °C overnight. After washing three times in phosphate‐buffered saline with Tween‐20 (PBST), the samples were incubated with secondary antibodies for 2–4 h at room temperature. The samples were counterstained with Hoechst for 10 min after rinsing for three times with PBST. The images were captured using a laser scanning confocal microscope (LSM 900, Carl Zeiss, Germany). Immunofluorescence images were analyzed and quantified by ZEN software. Colocalization analysis for Pearson's correlation coefficient and related fluorescence intensity quantification were performed using ImageJ software. The antibodies used in this study are listed in Table S3 of the Supporting Information.

### Detection of Nascent Protein Synthesis

To visualize nascent protein synthesis, Click‐iT HPG Alexa Fluor 594 Protein Synthesis Assay kit (Thermofisher scientific, USA) was used. Embryos were incubated in M2 medium containing 50 µm HPG for 1 h at 37 °C with 5% CO_2_. Fixation, permeabilization, and labeling were performed according to the manufacturer's protocol. After the Click‐iT reaction, embryos were subjected to immunofluorescence analysis as described above.

### 5‐Ethynyl Uridine (EU) Incorporation Assay

For the detection of transcription activity, embryos at different developmental stages were cultured in KSOM supplemented with 500 µm EU for 2 h at 37 °C with 5% CO_2_. Then the embryos were fixed with 4% PFA, permeabilized with 0.5% Triton X‐100 for 10 min, and stained with Cell‐Light EU Apollo 488 In vitro Imaging Kit (RiboBio, China) for 30 min according to the manufacturer's protocol. Embryos were then subjected to immunofluorescence analysis as described above.

### Western Blotting

Equal amounts of oocytes were collected and lysed using RIPA lysis buffer including protease inhibitors (Servicebio, China). Lysates were added to SDS loading buffer and incubated at 98 °C for 5 min before electrophoresis. Proteins were separated using SDS‐PAGE gel and then transferred to PVDF membranes. After that, membranes were blocked in a 5% nonfat milk solution for 1 h at room temperature and incubated with primary antibodies for 12 h at 4 °C. Following three washes with TBS Tween‐20 solution, the membranes were incubated with a secondary antibody for 2 h at room temperature. After washing for three times, the membranes were treated with ECL Western Blotting Substrate (Affinity Biosciences, China) and visualized using ChemiDoc XRS+ image system. The expression levels of each protein were normalized to GAPDH as an internal standard. The primary and secondary antibodies used were provided in Table S3 of the Supporting Information.

### Detection of Mitochondrial Localization and Mitochondrial Membrane Potential (MMP)

For mitochondrion staining, embryos were cultured in medium supplemented with cell‐permeant MitoTracker Red CMXRos (1:1000) (Beyotime, China) for 30 min in a humidified chamber at 37 °C with 5% CO_2_, respectively. After washing for three times with fresh M2 medium for 5 min each time, samples were imaged immediately under the laser scanning confocal microscope (LSM 900, Carl Zeiss, Germany). For MMP detection, embryos were cultured in M2 medium containing 2 µm JC‐1 (Beyotime, China) for 30 min in a humidified chamber at 37 °C with 5% CO_2_. After washing for three times with fresh M2 medium, samples were imaged immediately under the laser scanning confocal microscope. MMP was indicated by the ratio of red/green fluorescence intensity.

### Mitochondrial DNA (mtDNA) Copy Number Detection

For mtDNA extraction, 20 early embryos were added to 0.2 mL centrifuge tube containing 20 µL lysis buffer (0.5% Triton X‐100, 200 µg mL^−1^ proteinase K, 50 mm Tris‐HCl pH 8.0) and incubated at 55 °C for 2 h. Real‐time PCR was performed to detect the mtDNA copy number using ND1 and β‐globin primers. Primer sequences were provided in Table S1 of the Supporting Information.

### Low‐Input Quantitative RT‐PCR (qRT‐PCR)

For qRT‐PCR analysis, every 5–10 early embryos were lysed in 5 µL lysis buffer containing Reaction Mix and 0.1 µm Assay Pool. Reverse transcription was performed with the Single Cell Sequence Specific Amplification Kit (Vazyme, China) according to the manufacturer's procedure. qRT‐PCR was performed with SYBR green master mix (Yeasen, China) on Quantagene q225 qPCR system (Kubo Technology, Beijing) according to the manufacturers’ instructions. RNA levels of genes were normalized to *Gapdh*. The primer sequences for this study are listed in Table S1 of the Supporting Information.

### Low‐Input RNA Sequencing and Data Analysis

The RNA‐seq libraries of mouse early embryos were generated following the Smart‐Seq2 instruction as described previously with minor modification.^[^ ^40^ ^]^ Briefly, 10 embryos from each group were collected and washed six times with PBS and then immediately transferred into hypotonic lysis buffer. An Oligo‐dT primer was introduced to the reverse transcription reaction for first‐strand cDNA synthesis, followed by PCR amplification to enrich the cDNA. PCR amplification was performed to enrich cDNA, which was purified by AMPure XP beads, and checked using Qubit 3.0 Flurometer and Agilent 2100 Bioanalyzer. cDNA was then sheared by Covaris and subjected to Illumina TruSeq library preparation. The libraries were then qualified and loaded on Illumina Hiseq platform for PE150 sequencing (Annoroad Gene Technology, China).

Raw reads were processed with cutadapt v1.16 to remove adapters and perform quality trimming with default parameters. Trimmed reads were mapped to the mouse genome (mm10) using STAR (v2.5.3b) with default settings. The differential expression of genes was evaluated using the R package Deseq2 (v1.24.0). Genes were classified as differentially expressed as those which were more than twofold increased or decreased with *P*‐value < 0.05. Database for Annotation, Visualization and Integrated Discovery (DAVID, v2023q4) tools were used to analyze the enrichment of DEGs.^[^ ^41^ ^]^ The enrichment results containing three kinds of related functions of MF, biological process, and CC were obtained by using the function of GO enrichment analysis. By KEGG pathway enrichment analysis, the pathways and the related genes enriched in the pathways were derived, and the related pathways and genes with *p*‐value < 0.05 were analyzed. The enrichment results were visualized using R (v4.2.3). GSEA analyses were plotted by https://www.bioinformatics.com.cn, an online platform for data analysis and visualization.

### ChIP‐Seq Library Construction and Data Analysis

ChIP‐seq libraries were produced using a Hyperactive Universal CUT&Tag Assay Kit for Illumina (Vazyme, China) according to the manufacturer's protocol with anti‐Pol II antibody, anti‐H3K27ac antibody, and anti‐H3.3 antibody. A total of 30–60 early embryos were utilized for each library preparation. Generally, embryos without zona pellucidae were bound to equilibrated ConA Beads Pro for permeabilization, followed by the addition of the primary antibody and secondary antibody. Next, pA/G‐Tnp Pro was added to the samples for tagmentation. Finally, the libraries were purified with VAHTS DNA Clean Beads (Vazyme, China) and were send out for PE150 sequencing using Illumina HiSeq 3000 (Annoroad Gene Technology, China). The antibody information was provided in Table S3 of the Supporting Information.

Raw reads were processed with Trim Galore (v0.6.4) to remove adaptor sequences and poor‐quality bases with the specified parameters “‐q 20 ‐phred33 ‐stringency 5 ‐length 20 ‐paired”. Trimmed reads were then aligned to the mouse reference genome (mm10) using Bowtie2 (version 2.4.2) with default parameters. Picard (version 2.26.6) was preformed to remove PCR duplicates. Peaks were identified using MACS2 v2.2.7.1^[^ ^42^ ^]^ with default parameters. The bedgraph files were converted first to bigwig files using the UCSC Genome Browser tool bedGraphToBigWig^[^ ^43^ ^]^ and then visualized in the Integrative Genomics Viewer. Density plot of signal enrichment was generated by bamCoverage, computeMatrix, and plotHeatmap of the Python package deepTools (v3.5.1).^[^ ^44^ ^]^ ChIP‐seq peak annotations were conducted using the R package ChIPseeker (v1.34.1).^[^ ^45^ ^]^

### Low‐Input Whole Genome Bisulfite Sequencing (WGBS)

A total of 5–10 8‐cell embryos were collected from control, MFN1‐knockdown (KD) or H3.3‐KD groups. The WGBS library was prepared using a post‐bisulfite adapter tagging (PBAT) approach.^[^ ^46^ ^]^ EZ DNA Methylation‐Gold Kit (Zymo, USA) was performed for samples bisulfite conversion. Paired‐end reads for each sample was generated by PE150 sequencing using Illumina HiSeq 3000 (Annoroad Gene Technology, China).

Raw reads were processed with Trim Galore (v0.6.4) to discard adaptor sequences and poor‐quality bases with the parameter “‐q 20 ‐phred33 ‐stringency 5 ‐length 20 ‐paired”. Trimmed reads were then mapped to the mouse reference genome (mm10) using Bismark (v0.22.3) with the parameters “‐p 6 ‐parallel 1 ‐N 0 ‐L 20 ‐quiet ‐pbat ‐un ‐ambiguous ‐bam”. SAMtools (v1.3.1) was preformed to sort bam files by genomic coordination and establish a bam file index. Picard (v2.23.3) was carried out to remove PCR duplicates. The methylation level at each CpG site was calculated using bismark_methylation_extractor model with the parameters “‐p ‐comprehensive ‐no_overlap ‐bedgraph ‐counts ‐report ‐cytosine_report ‐gzip ‐buffer ‐size 30G”. The R package methylKit (v1.14.2)^[^ ^47^ ^]^ was used to identify DMRs. The methylation levels at CpG sites were firstly calculated by “methRead” function with mincov = 3. Methylation across the genome was tiled with the “tileMethylCounts” function using the parameters “win.size = 500, step. size = 500, cov. bases = 5”, then “unite” function was used to unite tiled regions with the “destrand = TRUE” parameter. At last, “calculateDiffMeth” function was used to calculate DMRs. DMRs with a minimum of 3 CpG sites and absolute methylation mean difference >10% and *q*‐value < 0.05 were used for further analysis. The UCSC Genome Browser utility,^[^ ^43^ ^]^ bedGraphToBigWig, was used to transform the bedgraph files to bigwig files. Next, UCSC genome browser was employed for visualization.

### Statistical Analysis

Statistical analyses were performed by R or GraphPad Prism 8.0 software (GraphPad, San Diego, USA). Quantifications are presented as mean ± SEM. Statistical comparison was performed using two‐tailed Student's *t*‐tests and Mann–Whitney *U* test. *P* < 0.05 was considered as statistically significant (*p < 0.05, **p < 0.01, and ***p < 0.001).

## Conflict of Interest

The authors declare no conflict of interest.

## Author Contributions

L.‐q.Z., B.‐x.M., and X.H. conceived, designed, and supervised the study. X‐.y.S. performed the experiments. Y.T., Y.‐f.W., and Q.T. helped with the experiments. Y.T., Y.Y., and X.H. performed high‐throughput data analysis. B.‐x.M. collected the human embryos and helped with the experiments. L.L. helped with the experimental design and provided valuable guidance. X.‐y.S. wrote the initial manuscript. L.‐q.Z. revised the manuscript. All authors contributed to the article and approved the manuscript.

## Supporting information



Supporting Information

## Data Availability

The RNA‐seq, ChIP‐seq, and DNA methylome datasets in this study have been deposited to NCBI: GSE276709, GSE276708, and GSE276710.
