# Supplementary material for: Mitofusin 1 Drives Preimplantation Development by Enhancing Chromatin Incorporation of Histone H3.3
Source: Adv Sci (Weinh). 2025 Mar 16;12(18):2414985. doi: 10.1002/advs.202414985 (PMC12079336; doi:10.1002/advs.202414985)
Supplement: Supplementary file 1 — Supporting Information [file ADVS-12-2414985-s001.pdf]

ADVANCED SCIENCE
Open Access

Supporting Information

for *Adv. Sci.*, DOI 10.1002/advs.202414985

Mitofusin 1 Drives Preimplantation Development by Enhancing Chromatin Incorporation of Histone H3.3

Xiao-yan Shi, Yu Tian, Yu-fan Wang, Yi-ran Zhang, Ying Yin, Qing Tian, Lei Li, Bing-xin Ma, Ximiao He* and Li-quan Zhou**

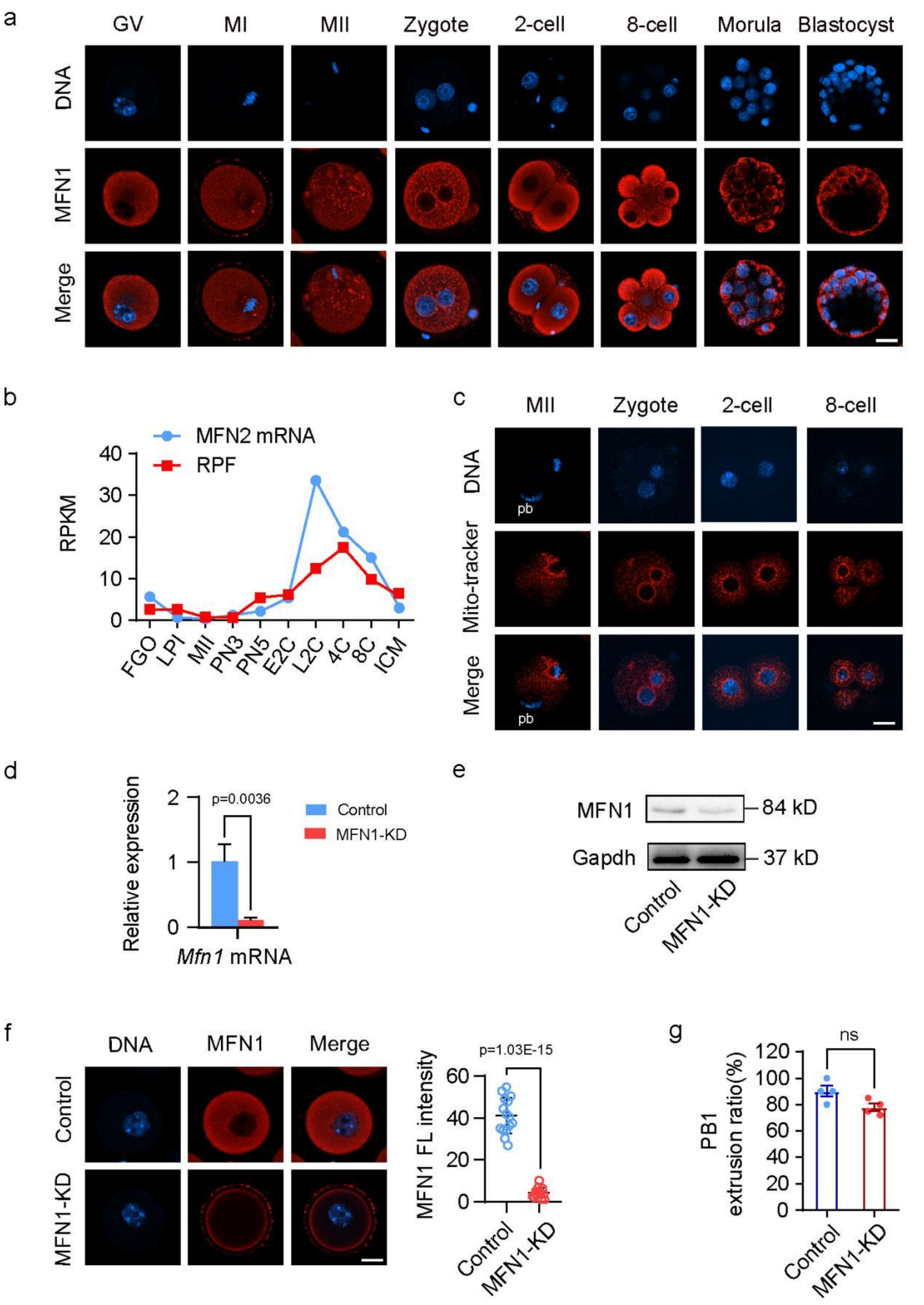

Fig. S1 The expression of MFN1 in mouse oocyte and early embryo and knockdown efficiency.

a Immunostaining of MFN1 at different developmental stages. GV (n = 50); MI (n = 51); MII (n = 55); 1-cell (n = 68); 2-cell (n = 60); 8-cell (n = 70); morula (n = 68); blastocyst (n = 73). Scale bar, 25 μm.

b The dynamics of *Mfn2* transcript (mRNA-seq signal) and translation (Ribo-seq

signal, RPF, representing the efficiency of translation) levels. Data are expressed as the mean of RPKM from two repeats. RPKM, reads per kilobase of bin per million mapped reads; FGO, LPI, and MII, full-grown, late prometaphase I and MII oocytes; PN3 and PN5, PN3 and PN5 stage of early1-cell embryos; E2C and L2C, early and late 2-cell embryos; 4C and 8C, 4- and 8-cell embryos; ICM, inner cell mass. Public dataset GSE165782 was used for analysis.

c Immunostaining of mitochondrial distribution in MII, zygotes, 2-cell and 8-cell embryos from control (n = 22, n = 23, n = 20, n = 19) and MFN1-KD (n =18, n = 25, n = 26, n = 25) groups. Scale bar, 25 µm.

d qRT-PCR comparing relative expression levels of *Mfn1* mRNA in control and MFN1-KD GV oocytes. The expression levels were normalized to *Gapdh*, which served as an internal control.

e Western blot comparing MFN1 protein levels in control and MFN1-KD GV oocytes. *Gapdh* is the internal control.

f Immunofluorescence staining (left) and the fluorescence intensities (right) of MFN1 in control (n = 15) and MFN1-KD (n = 15) GV oocytes. Scale bar, 25 µm.

g Quantification of PB1 extrusion rate in control and MFN1-KD oocytes. n = 4 biological replicates. ns, not significant.

Data of (**d**), (**f**) and (**g**) were presented as mean ± SEM of at least three independent experiments. P value was calculated using two-tailed Student's t test.

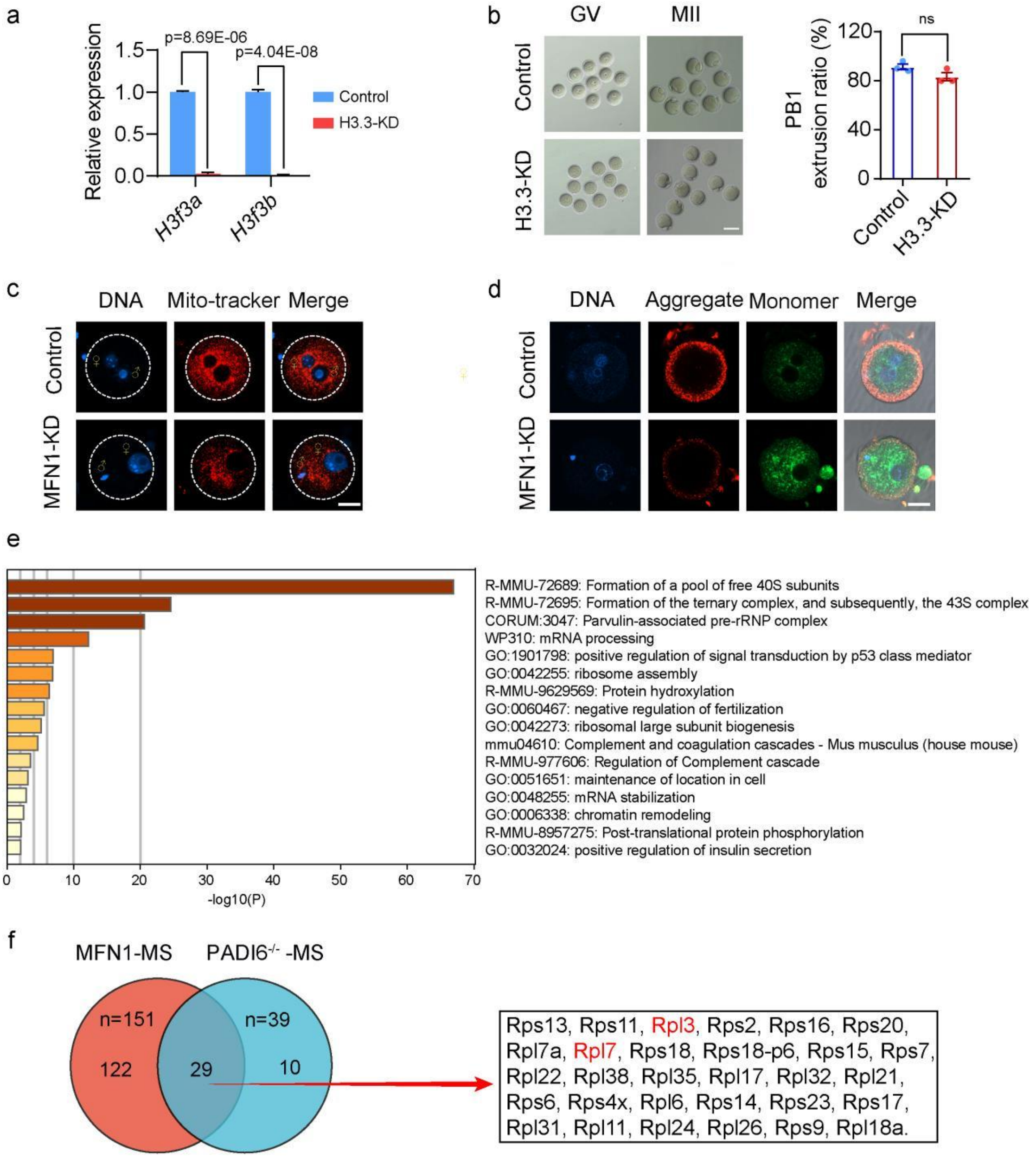

Fig. S2 The impairment of mitochondrial function by MFN1 depletion and the analysis of MFN1 coIP-MS results.

a qRT-PCR comparing relative expression levels of *H3f3a* and *H3f3b* mRNA between control and H3.3-KD GV oocytes. The expression levels were normalized to *Gapdh*, which served as an internal control.

b Representative DIC images (left) of GV and MII oocytes from control (n = 106) and H3.3-KD (n = 117) groups. GV oocytes were microinjected with siRNA against H3f3a and H3f3b. After 14 h, PB1 extrusion rates (right) were analyzed. Scale bar, 25 μm.

c-d Immunostaining of mitochondrial distribution (**c**) and JC1 staining (**d**) in zygotes from control and MFN1-KD groups. Scale bar, 25 μm.

e Gene ontology analysis of MS results using anti-MFN1 antibody in mouse oocytes by Metascape.

f The Venn diagram shows 29 common ribosomal subunits in IP-MS result of MFN1 protein and published MS data from PADI6$^{-/-}$ oocytes.

Data of (**a**) and (**b**) were presented as mean ± SEM from at least three independent experiments. P value was calculated by two-tailed Student's t-test. ns, not significant.

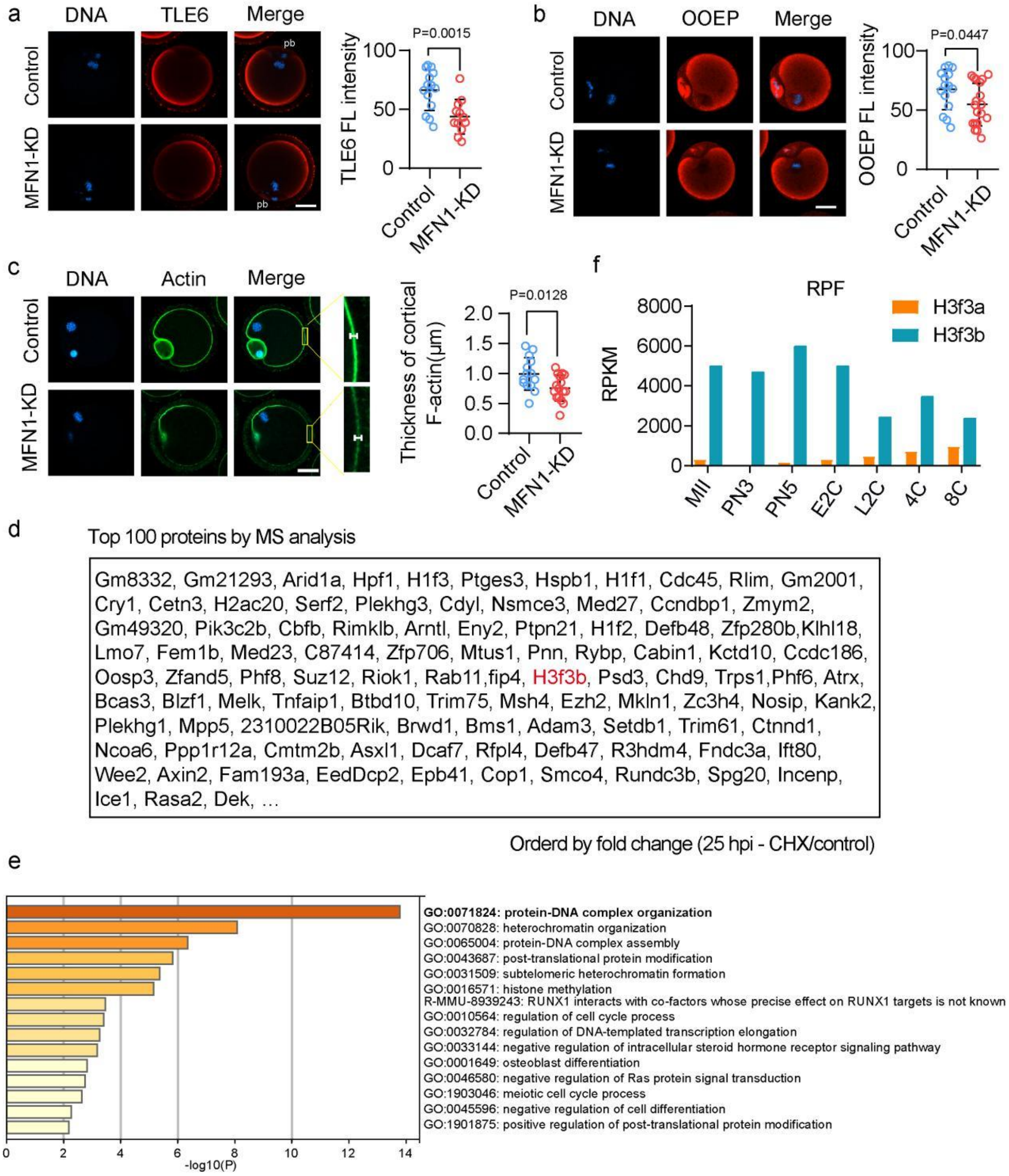

Fig. S3 The impairment of MFN1 on components of cytoplasmic lattice and the proteins sensitive to translation inhibition.

a Representative image of immunofluorescence staining for TLE6 in control and MFN1-KD MII oocytes (left). Scale bar, 25 μm. Quantification signals of TLE6 in control and MFN1-KD MII oocytes (right). Control (n = 14), MFN1-KD (n = 12).

b Representative image of immunofluorescence staining for OOEP in control and

MFN1-KD MII oocytes (left). Scale bar, 25 μm. Quantification signals of OOEP in control and MFN1-KD MII oocytes (right). Control (n = 15), MFN1-KD (n = 19).

c Representative image of immunofluorescence staining for F-actin in control (n = 14) and MFN1-KD (n = 16) MII oocytes (left). The right is an enlarged inset image. Scale bar, 25 μm. Quantification of cortical F-actin width in control and MFN1-KD MII oocytes (right).

d The top 100 proteins with high translation activity, ordered by fold change (25hpi-CHX/control, hpi, hours post-insemination).

e Gene ontology analysis of (**d**).

f The dynamics of *H3f3a* and *H3f3b* translation activity (Ribo-seq signal, RPF, representing the efficiency of translation). Public dataset GSE165782 was used for analysis**.**

Data of (**a**), (**b**) and (**c**) were presented as mean ± SEM from at least three independent experiments. P value was calculated by two-tailed Student's t-test. ns, not significant.

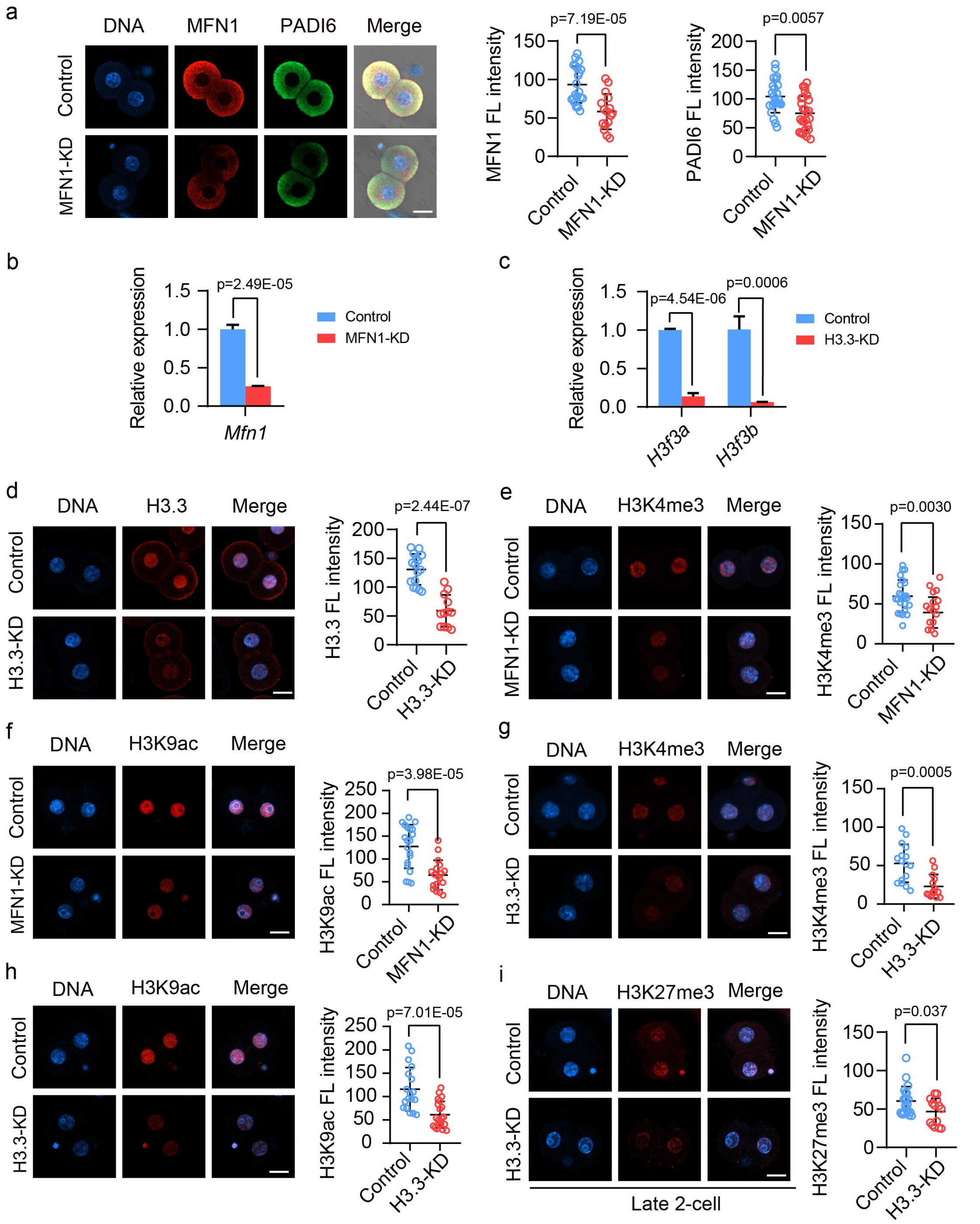

Fig. S4 The effect of MFN1 or H3.3 knockdown on histone modifications in 2-cell embryos.

a Representative images of MFN1 and PADI6 localization in control and MFN1-KD 2-cell embryos (left). Scale bar, 25 μm. The fluorescence intensities of MFN1 and PADI6 signals were examined in control (n = 21) and MFN1-KD (n = 16) early 2-cell embryos (right).

b The depletion efficiency of MFN1 at the levels of mRNA (**b**) and protein (**c**) in early 2-cell embryos by qRT-PCR and immunostaining . The mRNA expression levels were normalized to *Gapdh*, which served as an internal control.

c, d The depletion efficiency of H3.3 at the levels of mRNA (**c**) and protein as shown

with immunostaining (**d**) in early 2-cell embryos. The mRNA expression levels were normalized to *Gapdh*, which served as an internal control. In (**d**), control (n = 16) and MFN1-KD (n = 12). Scale bar, 25 μm.

e Immunostaining images (left) and quantification signals (right) of H3K4me3 for mouse early 2-cell embryos from control (n = 24) and MFN1-KD (n = 16) groups. Scale bar, 25 μm.

f Immunostaining images (left) and quantification signals (right) of H3K9ac for mouse early 2-cell embryos from control (n = 20) and MFN1-KD (n = 18) groups. Scale bar, 25 μm.

g Immunostaining images (left) and quantification signals (right) of H3K4me3 for mouse early 2-cell embryos from control (n = 16) and H3.3-KD (n = 14) groups. Scale bar, 25 μm.

h Immunostaining images (left) and quantification signals (right) of H3K9ac for mouse early 2-cell embryos from control (n = 18) and H3.3-KD (n = 21) groups. Scale bar, 25 μm.

i Immunostaining images (left) and quantification signals (right) of H3K27me3 for mouse late 2-cell embryos from control (n = 24) and H3.3-KD (n = 26) groups. Scale bar, 25 μm.

Data of (**a-i**) were presented as mean ± SEM of at least three independent experiments. P value was performed by two-tailed Student's t test.

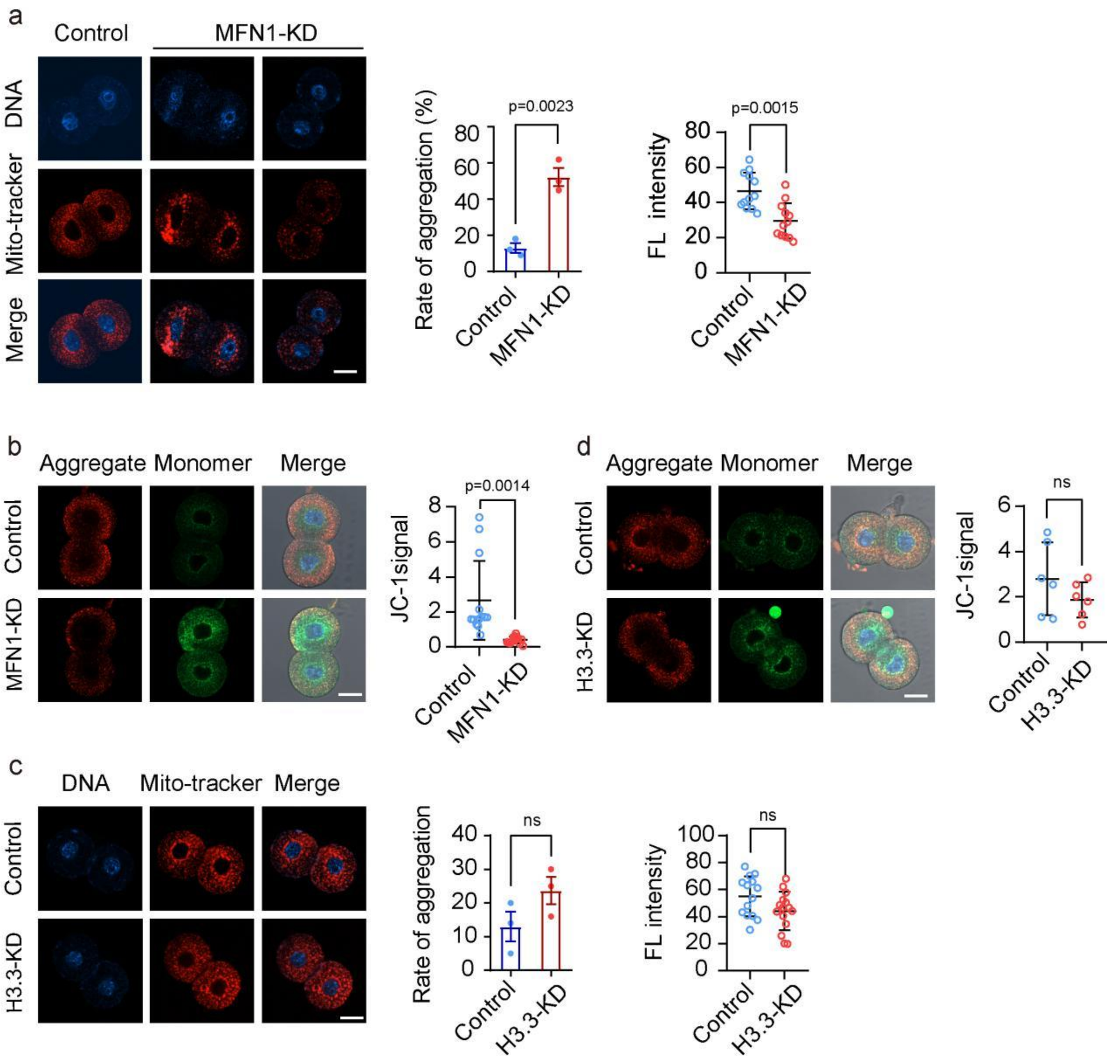

Fig. S5 The impact of MFN1 and H3.3 depletion on mitochondrial distribution and function in early 2-cell embryos.

a Early 2-cell embryos with *Mfn1* depletion were stained with Mito-tracker Red to show mitochondrial distribution (left panel). Scale bar, 25μm. Quantification of aggregation rate and fluorescence intensity of Mito-tracker signals in control (n = 12) and MFN1-KD (n = 12) early 2-cell embryos (right panel).

b Representative images (left panel) of MMP in control (n = 13) and MFN1-KD (n = 13) early 2-cell embryos. Red, high membrane potential; green, low membrane potential. Scale bar, 25 μm. The ratio of red to green fluorescence intensity was quantified in control and MFN1-KD early 2-cell embryos (right panel).

c Early 2-cell embryos with H3.3 depletion were stained with Mito-tracker Red to show mitochondrial distribution (left panel). Scale bar, 25μm. Quantification of aggregation rate and fluorescence intensity of Mito-tracker signals in control (n = 14) and H3.3-KD (n = 15) early 2-cell embryos (right panel).

d Representative images (left panel) of MMP in control (n = 6) and H3.3-KD (n = 6) early 2-cell embryos. Red, high membrane potential; green, low membrane potential. Scale bar, 25 μm. The ratio of red to green fluorescence intensity was quantified in control and H3.3-KD early 2-cell embryos (right panel).

Data of (**a-d**) were presented as mean ± SEM of at least three independent experiments. P value was performed by two-tailed Student's t test.

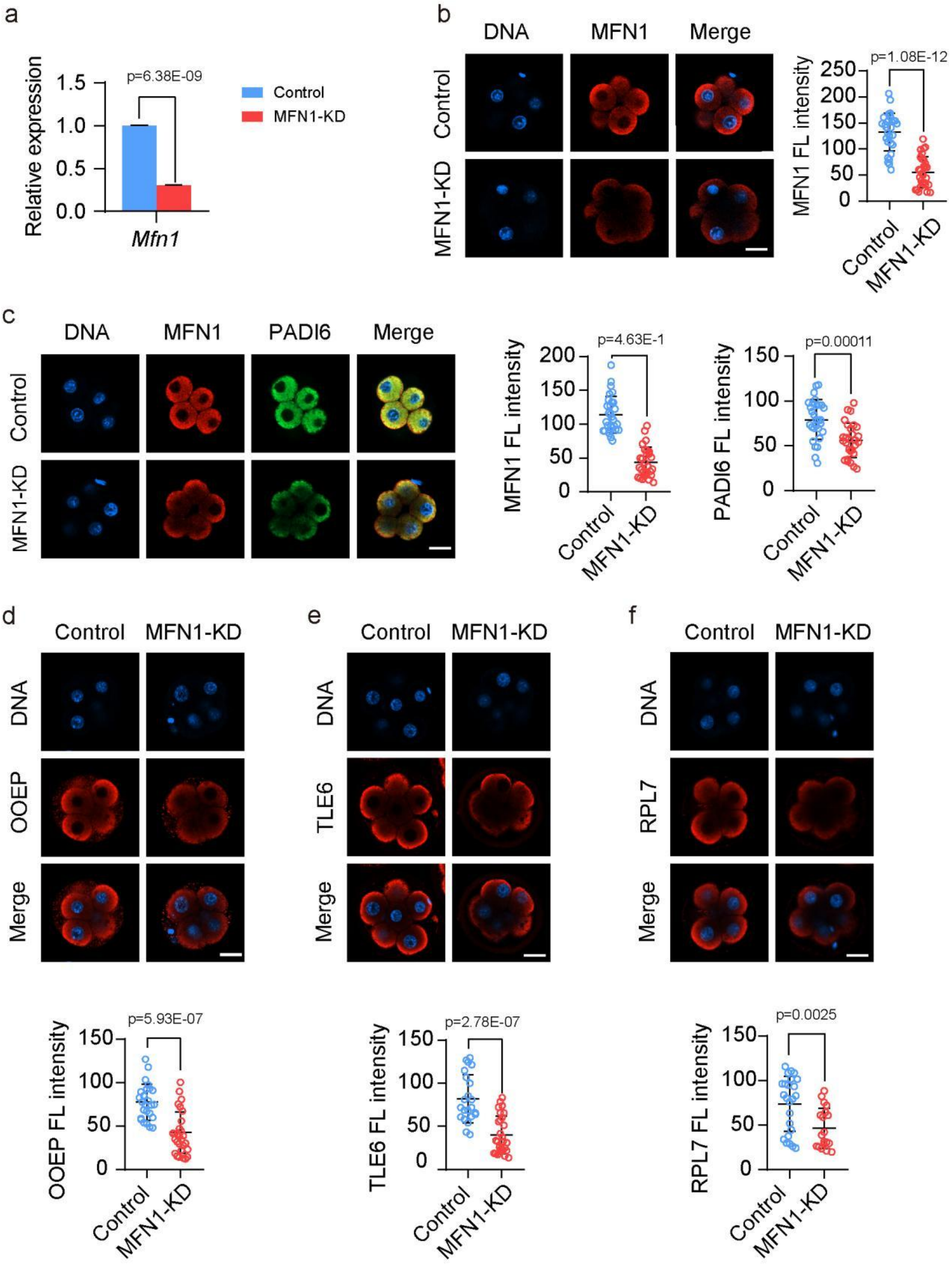

Fig. S6 Effects of MFN1 knockdown on the components of cytoplasmic lattices and ribosomal proteins in 8-cell embryos.

a, b The depletion efficiency of MFN1 at the levels of mRNA (**a**) and protein as shown with immunostaining (**b**) in 8-cell embryos. Scale bar, 25 µm. In (**b**), control (n = 30) and MFN1-KD (n = 30).

c Representative images of MFN1 and PADI6 localization in control and MFN1-KD 8-cell embryos (left panel). 8-cell embryos were immunostained with anti-MFN1 and anti-PADI6 antibodies and imaged by confocal microscope with identical settings and parameters. Scale bar, 25 µm. The fluorescence intensity of MFN1 and PADI6 signals was recorded in control (n = 29) and MFN1-KD (n = 28) 8-cell embryos (right panel).
d Representative images of OOEP in control and MFN1-KD 8-cells embryos (upper panel). Scale bar, 25 µm. The fluorescence intensity of OOEP signals was measured in control (n = 25) and MFN1-KD (n = 28) 8-cell embryos (lower panel).
e Representative images of TLE6 in control and MFN1-KD 8-cells embryos (upper panel). Scale bar, 25 µm. The fluorescence intensity of TLE6 signals was recorded in control (n = 22) and MFN1-KD (n = 28) 8-cell embryos (lower panel).
f Representative images of RPL7 in control and MFN1-KD 8-cells embryos (upper panel). Scale bar, 25 µm. The fluorescence intensity of RPL7 signals was measured in control (n = 24) and MFN1-KD (n = 19) 8-cell embryos (lower panel).
Data of (**a-f**) were presented as mean ± SEM of at least three independent experiments. P value was performed by two-tailed Student's t test.

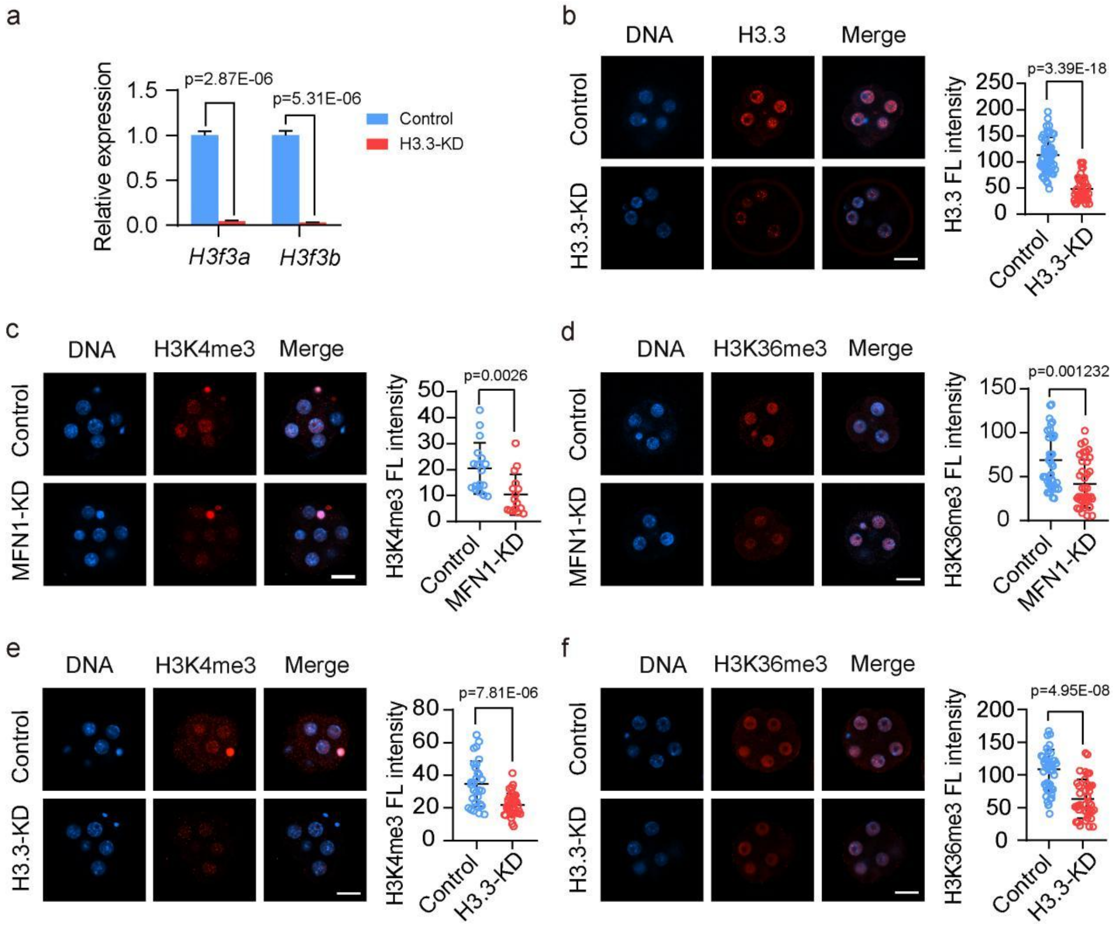

Fig. S7 The effect of MFN1 or H3.3 knockdown on histone modifications in 8-cell embryos.
a, b The depletion efficiency of H3.3 at the levels of mRNA (**a**) and protein as shown with immunostaining (**b**) in 8-cell embryos. Scale bar, 25 µm. In (**b**), control (n = 54)

and H3.3-KD (n = 43).

c Immunostaining images (left panel) and quantification signals (right panel) of H3K4me3 for 8-cell embryos from control (n = 17) and MFN1-KD (n = 16) groups. Scale bar, 25 μm.

d Immunostaining images (left panel) and quantification signals (right panel) of H3K36me3 for mouse 8-cell embryos from control (n = 39) and MFN1-KD (n = 39) groups. Scale bar, 25 μm.

e Immunostaining images (left panel) and quantification signals (right panel) of H3K4me3 for mouse 8-cell embryos from control (n = 30) and H3.3-KD (n = 36) groups. Scale bar, 25 μm.

f Immunostaining images (left panel) and quantification signals (right panel) of H3K36me3 for mouse 8-cell embryos from control (n = 45) and H3.3-KD (n = 40) groups. Scale bar, 25 μm.

Data of (**a-f**) were presented as mean ± SEM of at least three independent experiments. P value was performed by two-tailed Student's t test.

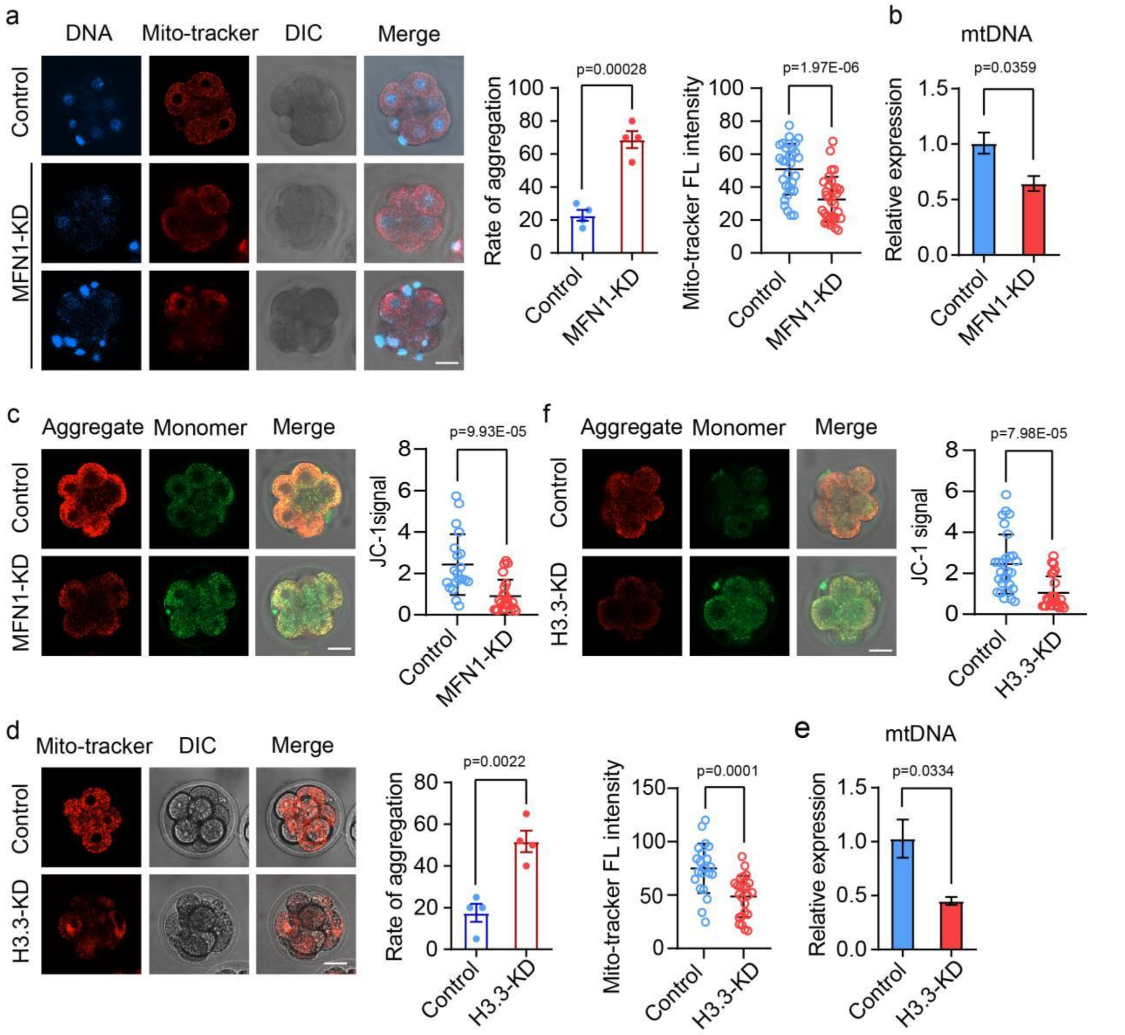

Fig. S8 Effects MFN1 and H3.3 knockdown on mitochondrial distribution and function in 8-cell embryos.

a Representative images of mitochondrial localization in control and MFN1-KD

8-cell embryos (left panel). Scale bar, 25 μm. The aggregation rate of mitochondrial distribution and the fluorescence intensity of Mito-tracker signals was measured in control (n = 31) and MFN1-KD (n = 31) 8-cell embryos (right panel).

b The mtDNA levels were measured in control and MFN1-KD 8-cell embryos. n = 4 biological replicates.

c MMP was detected by JC-1 staining (left panel) in control and MFN1-KD 8-cell embryos (Red, high membrane potential; green, low membrane potential). Scale bar, 25 μm. The ratio of red and green fluorescence intensity was calculated in control (n = 20) and MFN1-KD (n = 23) 8-cell embryos (right panel).

d Representative images of mitochondrial localization in control and H3.3-KD 8-cell embryos (left panel). Scale bar, 25 μm. The aggregation rate of mitochondrial distribution and the fluorescence intensity of Mito-tracker signals was measured in control (n = 22) and H3.3-KD (n = 24) 8-cell embryos (right panel).

e The mtDNA levels were measured in control and H3.3-KD 8-cell embryos. n = 3 biological replicates.

f MMP was detected by JC-1 staining (left panel) in control and H3.3-KD 8-cell embryos. Scale bar, 25 μm. The ratio of red and green fluorescence intensity was calculated in control (n = 28) and H3.3-KD (n = 25) 8-cell embryos (right panel).

Data of (**a-f**) were presented as mean ± SEM of at least three independent experiments. P value was performed by two-tailed Student's t test.

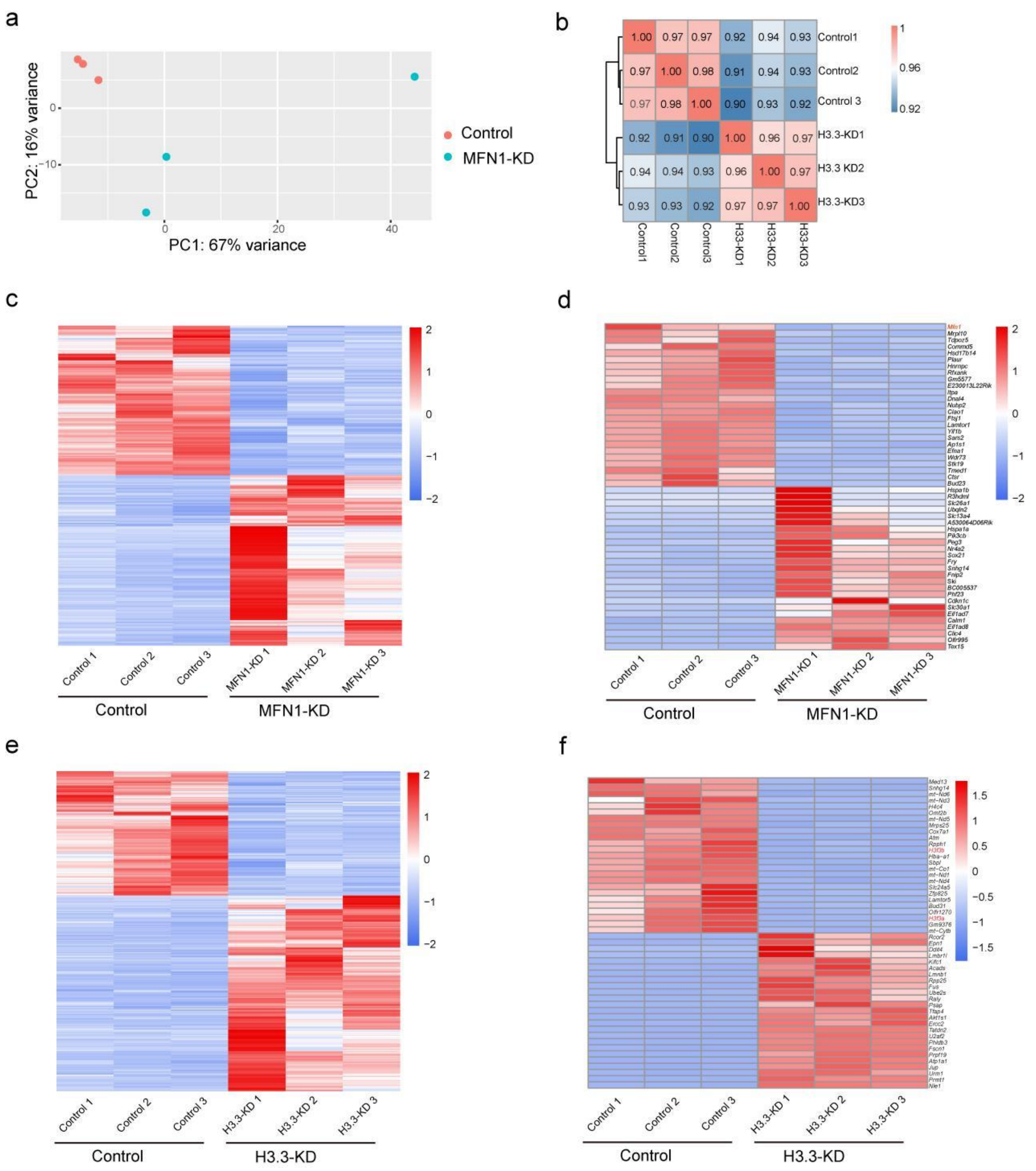

Fig. S9 Effects of MFN1 and H3.3 depletion on the transcriptome of 8-cell embryos.

a Principal Component Analysis (PCA) for transcriptomes of control and MFN1-KD 8-cell embryos.

b Heatmap of Pearson correlation coefficients for transcriptomes of control and H3.3-KD 8-cell embryos.

c Heatmap illustration displayed DEGs between control and MFN1-KD 8-cell embryos.

d Heatmap of top 50 DEGs of MFN1-KD 8-cell embryos by adjusted P values.

e Heatmap illustration showed DEGs between control and H3.3-KD 8-cell embryos.

f Heatmap of top 50 DEGs in H3.3-KD 8-cell embryos by adjusted P values.

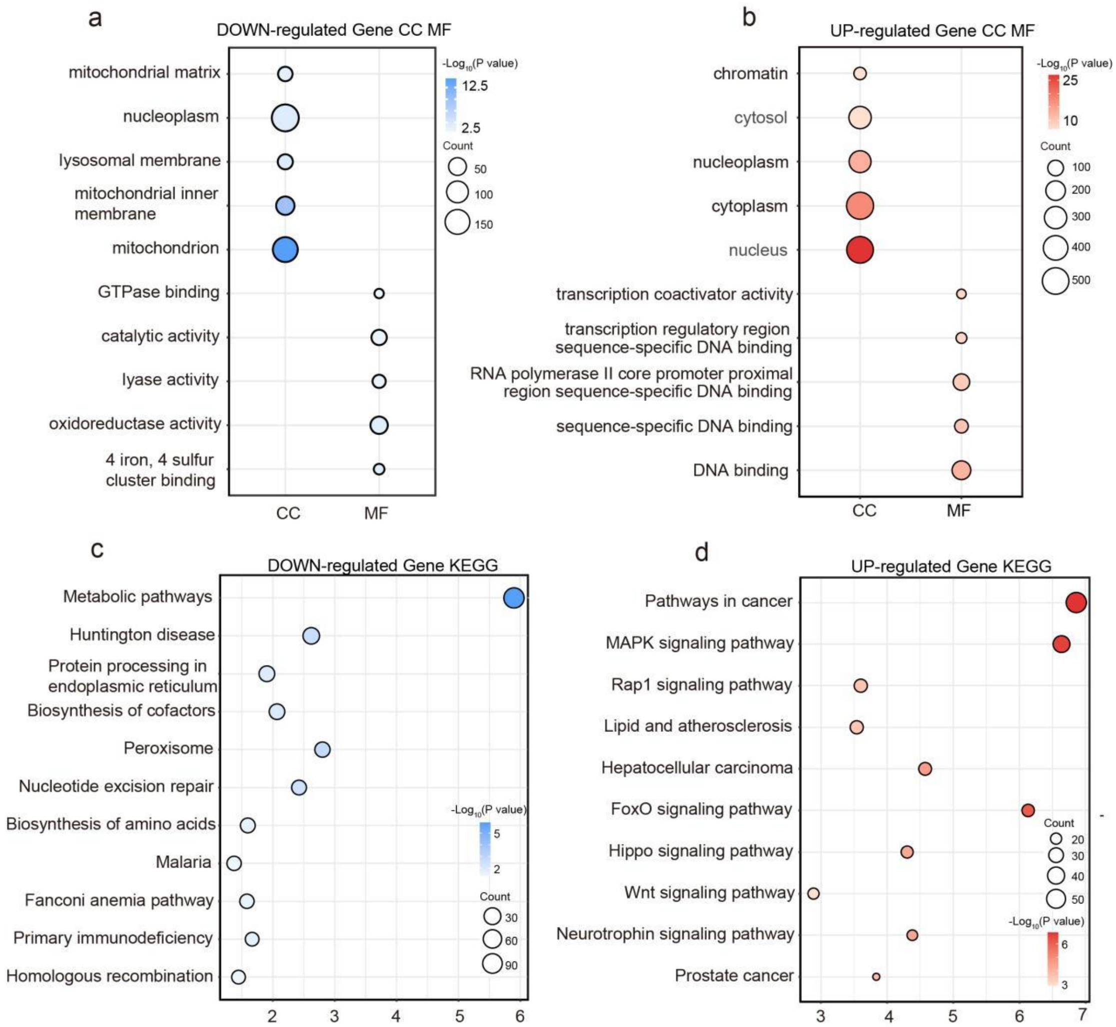

Fig. S10 Effect of MFN1 depletion on transcriptome of 8-cell embryos.

a-b GO enrichment analysis of downregulated (**a**) and (**b**) upregulated genes in MFN1-KD 8-cell embryos on cellular component and molecular function compared to control.

c-d KEGG enrichment analysis of downregulated (**c**) and upregulated (**d**) genes in MFN1-KD 8-cell embryos on cellular component and molecular function compared to control.

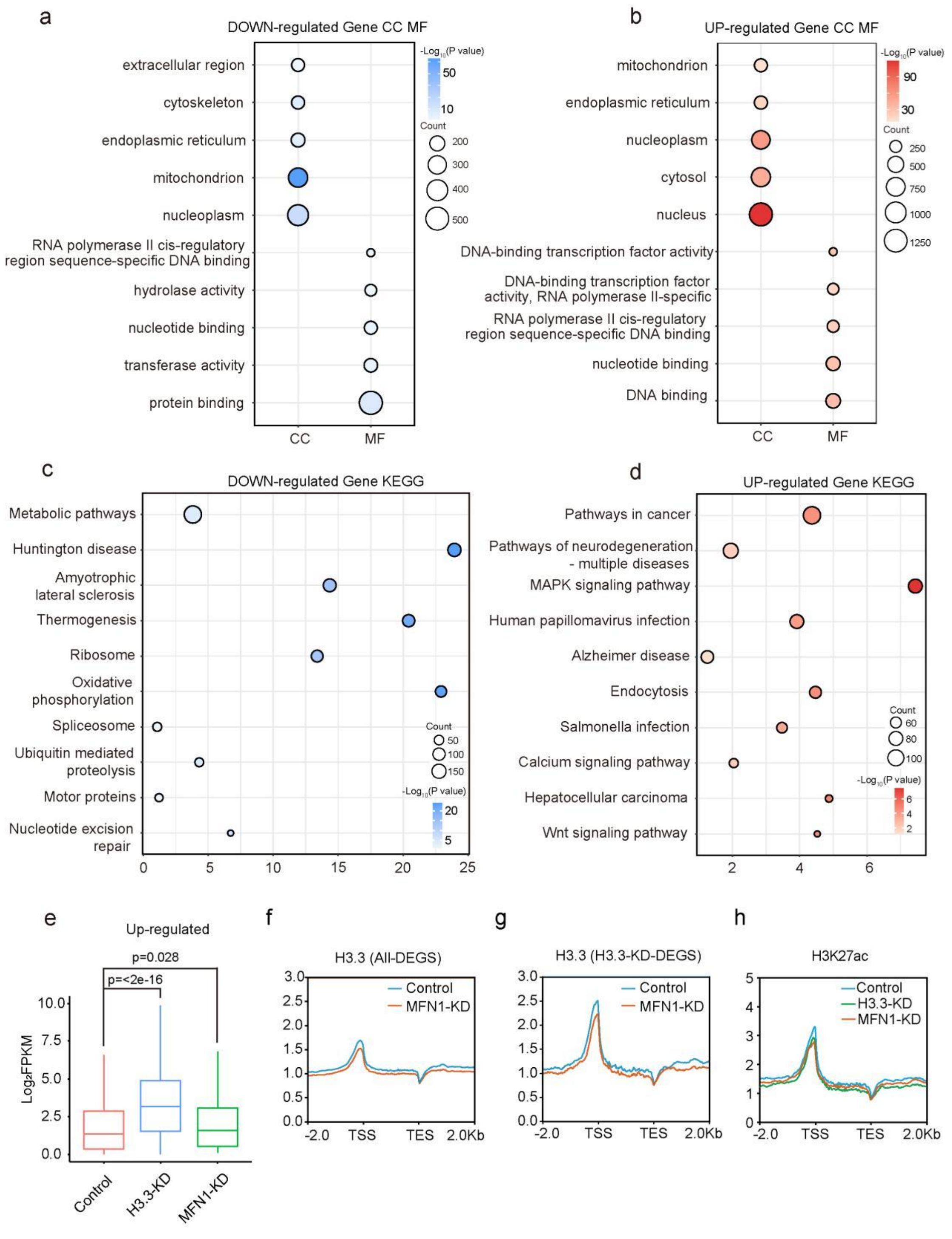

Fig. S11 Effect of H3.3 depletion on transcriptome of 8-cell embryos.

a-b GO enrichment analysis of downregulated (**a**) and (**b**) upregulated genes in H3.3-KD 8-cell embryos on cellular component and molecular function compared to control.

c-d KEGG enrichment analysis of downregulated (**c**) and upregulated (**d**) differentially expressed genes in H3.3-KD 8-cell embryos on cellular component and molecular function compared to control.

e Box plot of mRNA levels by RNA-seq depicting expression of upregulated genes by H3.3 knockdown in H3.3-KD and MFN1-KD 8-cell embryos. Mann-Whitney U test

was used to calculate P values.

f Density plot demonstrated the enrichment of H3.3 at 2 kb upstream/downstream of all genes in control and MFN1-KD 8-cell embryos.

g Density plot demonstrated the enrichment of H3.3 at 2 kb upstream/downstream of all DEGs by H3.3 depletion in control and MFN1-KD 8-cell embryos.

h Density plot of H3K27ac at 2 kb upstream/downstream of all genes in control, H3.3-KD and MFN1-KD 8-cell embryos.

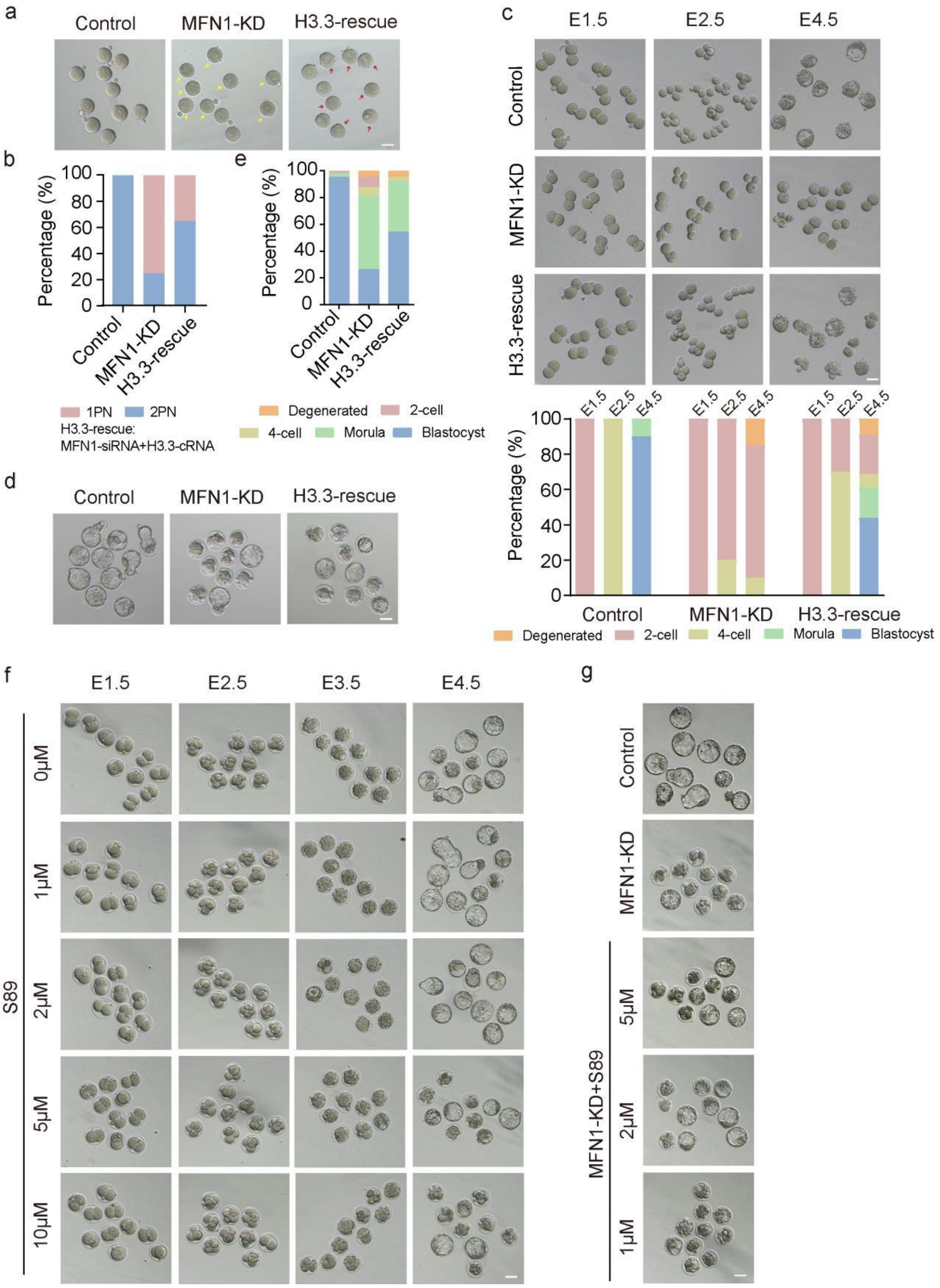

Fig. S12 Exogenous H3.3 and S89 supplementation exert rescuing effect on early embryonic developmental defects.

a-b Representative images (**a**) and stacked bar plots at the zygotes (**b**) of PN formation after fertilization in control oocytes (n = 96), MFN1-KD oocytes (n = 103), and co-injection of H3.3 cRNA into the MFN1-depleted oocytes (H3.3-rescue oocytes) (n = 105). GV oocytes from control group, MFN1-KD group, and H3.3-rescue group were cultured in M16 medium for *in vitro* maturation. Subsequently, matured oocytes

were selected for IVF, and the PN formation were analyzed. Compared with the MFN1-KD group, the proportion of 2PN formation in the H3.3-rescue group was significantly increased. Scale bar, 80 μm.

c Representative images (upper panel) and rates (lower bar graph) of early embryos at the indicated time points after fertilization in control (n = 80), MFN1-KD (n = 64), and H3.3-rescue groups (n = 66). Generally, MII oocytes were injected with control siRNA or siRNA against *Mfn1* before IVF. For the rescue experiment, H3.3 cRNA was co-injected with siRNA against *Mfn1*. Then, the developmental progression of early embryos in control, MFN1-KD, and H3.3-rescue groups was determined by recording the rate of early embryos at specific time points. The blastocyst rate in the H3.3-rescue group showed partial restoration. Scale bar, 80 μm.

d-e Representative images (d) and stacked bar plots (e) of blastocyst formation in control embryos (n = 107), MFN1-KD embryos (n = 112), and H3.3-rescue embryos (n = 110). Generally, after IVF, collected zygotes were injected with control siRNA or siRNA against *Mfn1*, followed by culturing in KSOM medium for early development examination. For the rescue experiment, H3.3 cRNA was co-injected with siRNA against *Mfn1*. The developmental progression of early embryos in control, MFN1-KD, and H3.3-rescue groups was determined by recording the rate of blastocyst at E4.5. The blastocyst rate in the H3.3-rescue group showed partial restoration. Scale bar, 80 μm.

f Representative images showing the developmental progression of early embryos with different concentrations of S89 (0 μM, 1 μM, 2 μM, 5 μM, and 10 μM) at each time point. Scale bar, 80 μm. 0 μM, n = 80. 1 μM, n = 80. 2 μM, n = 90. 5 μM, n = 90. and 10 μM, n = 85.

g Representative images of blastocyst formation in the control group, MFN1-KD group, and S89-rescue group with different concentrations (0 μM, 1 μM, 2 μM, and 5 μM). Scale bar, 80 μm. Control, n = 90. MFN1-KD, n = 88. 0 μM, n = 93. 1 μM, n = 86. 2 μM, n = 95. 5 μM, n = 87.

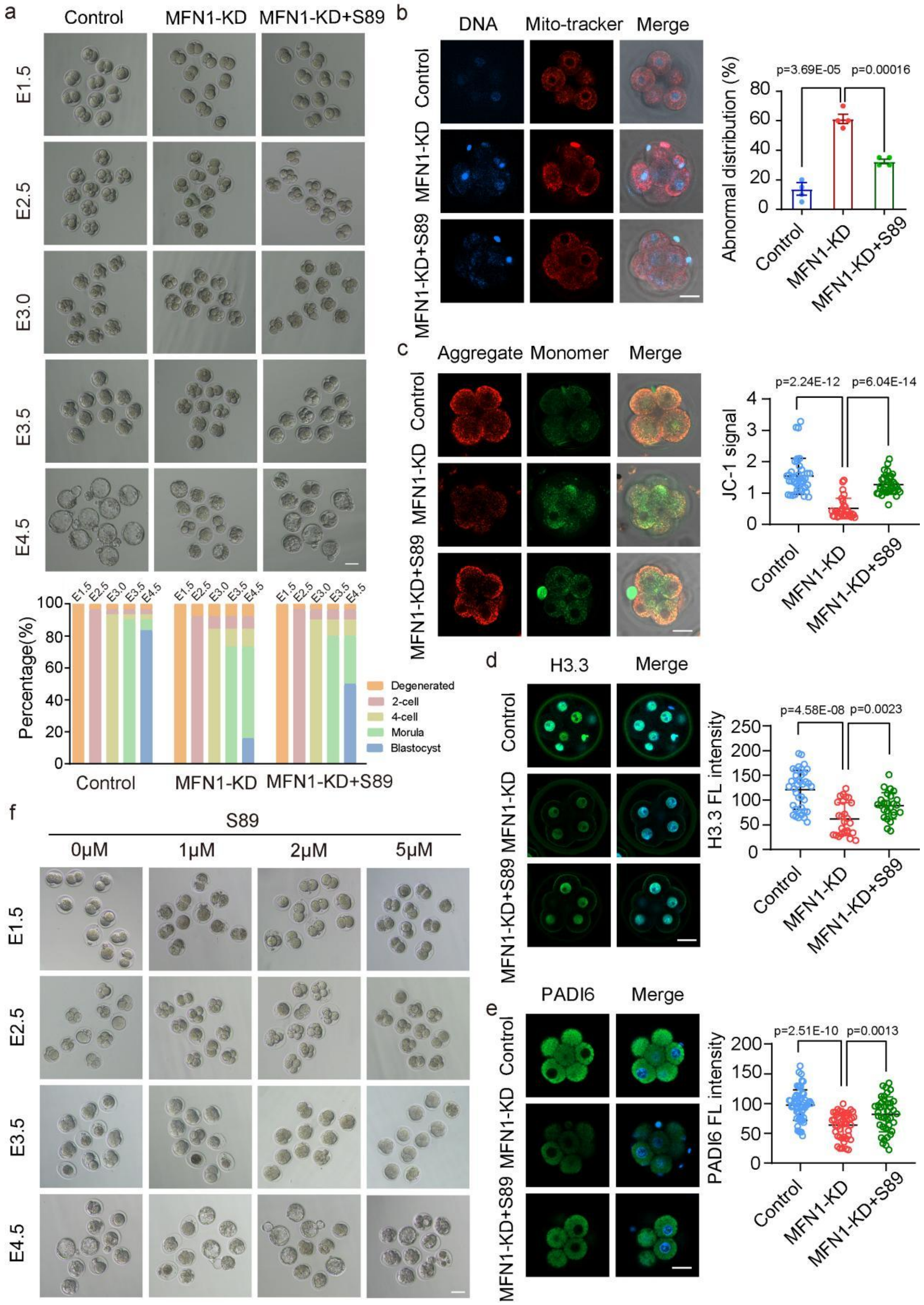

Fig. S13 Effects of S89 supplementation on early embryo development from MFN1-KD embryos and maternally aged embryos.

a Representative images (upper panel) showing the development of control (n = 90), MFN1-KD (n = 90) and MFN1-KD with S89 supplementation (n = 90) embryos at each time point. Scale bar, 80 μm. Quantification (lower panel) of the developmental rate at the indicated time points from at least three independent experiments.

b Mitochondrial distribution (left panel) and aggregation ratio (right panel) in 8-cell embryos from control (n = 53), MFN1-KD (n = 52) and MFN1-KD with S89 supplementation (n = 52) groups. Scale bar, 25 μm.
c JC1 staining (left panel) in control (n = 39), MFN1-KD (n = 29) and MFN1-KD with S89 supplementation (n = 35) 8-cell embryos to detect MMP. MMP level was indicated by red/green ratio (right panel). Scale bar, 25μm.
d Immunostaining (left panel) and fluorescence intensities (right panel) of H3.3 in control (n = 37), MFN1-KD (n = 26) and MFN1-KD with S89 supplementation (n = 25) 8-cell embryos. Scale bar, 25 μm.
e Immunofluorescence staining (left panel) and fluorescence intensities (right panel) of PADI6 in control (n = 52), MFN1-KD (n = 55) and MFN1-KD with S89 supplementation (n = 58) 8-cell embryos. Scale bar, 25 μm.
f Representative images illustrate the development of early embryos from aged female mice treated with different concentrations (0 μM, 1 μM, 2 μM, and 5 μM) of S89 at each time-point. 0 μM, n = 63. 1 μM, n = 65. 2 μM, n = 72. 5 μM, n = 69. Scale bar, 80 μm.
Data of (**b-e**) were presented as mean ± SEM from at least three independent experiments. P value was calculated by two-tailed Student's t-test.

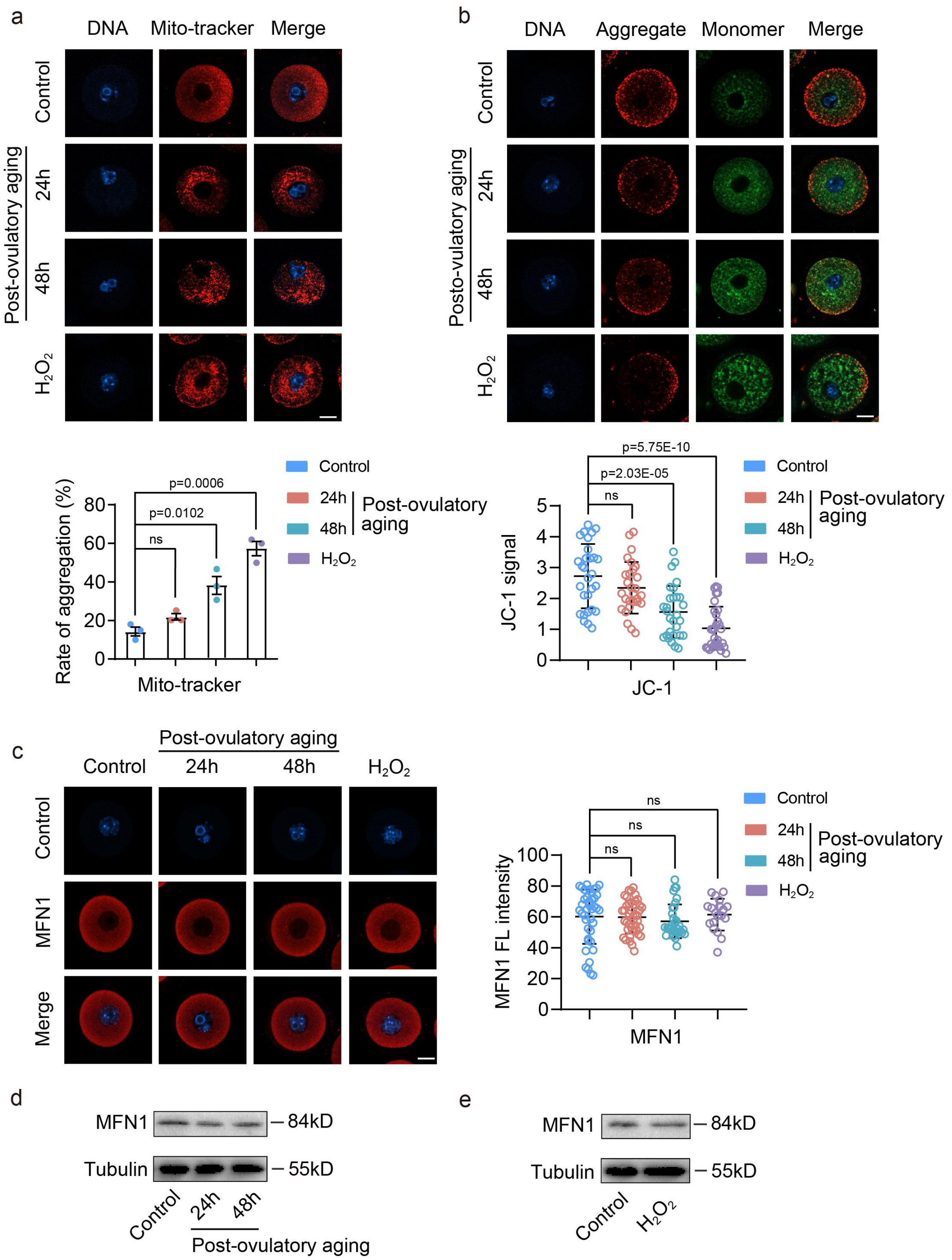

Fig. S14 The impact of *in vitro* simulated aging conditions on mitochondrial integrity and MFN1 proteins in mouse oocytes.

a Mitochondrial distribution (upper panel) and aggregation ratio (lower panel) in GV oocytes from control (n = 50), 24h of post-ovulatory aged (n = 48), 48h of post-ovulatory aged (n = 50) and H_2O_2 treated (n = 55) groups. Generally, GV oocytes were maintained for 24h or 48h in 2.5 μM milrinone for post-ovulatory aging treatment. For the H_2O_2-treated experiments, GV oocytes were cultured in 100 μM H_2O_2 for 30 minutes. Scale bar, 20 μm.

b JC1 staining (upper panel) in GV oocytes from control (n = 58), 24h of post-ovulatory aged (n = 55), 48h of post-ovulatory aged (n = 56) and H_2O_2-treated (n = 61) groups to detect MMP. MMP level was indicated by red/green ratio (lower panel). Scale bar, 20μm.
c Immunostaining (left panel) and fluorescence intensities (right panel) of MFN1 in control (n = 78), 24h of post-ovulatory aged (n = 85), 48h of post-ovulatory aged (n = 82) and H_2O_2-treated (n = 94) GV oocytes. Scale bar, 20 μm.
d Western blot comparing MFN1 protein levels in control and post-ovulatory aged GV oocytes. Tubulin was used as the internal control.
e Western blot comparing MFN1 protein levels in control and H_2O_2-treated GV oocytes. Tubulin was used as the internal control.
Data of (a, b, c) were presented as mean±SEM from at least three independent experiments. P value was calculated by two-tailed Student's t-test.

Supplementary Information

Table S1. The primer sequences used in this study

Primer	Sequence (5’-3’)
pVax1-IVT-F	CGTGTACGGTGGGAGGTCTA
pVax1-IVT-R	TTCGCTTGCTGTCCATAAAA
Zscan4-F	GAGATTCATGGAGAGTCTGACTGA
Zscan4-R	GCTGTTGTTTCAAAAGCTTGATGACTTC
Zfp352-F	ACCACCTCAAAGAACACCAG
Zfp352-R	ACAAGGGACAAGCGTAGAAC
MERV-L-F	ATCTCCTGGCACCTGGTATG
MERV-L-R	AGAAGAAGGCATTTGCCAGA
Tcstv1-F	GGATCCCTGAAGGTAAATCCTC
Tcstv1-R	AACCATCCATCCTCAGGAAC
Tdpoz4-F	ACCCAAGACCTGCAATCAAG
Tdpoz4-R	ATTCATGGCCAGCTACCAAC
Eifla-F	CCAAAGAATAAAGGCAAAGGAG
Eifla-R	CTCACACCGTCAAAGCACATT
Gapdh-F	TCTTCCAGGAGCGAGACCC
Gapdh-R	CGGAGATGATGACCCTTTT
5.8S F	CTTAGCGGTGGATCACTCGG
5.8S R	ACGCTCAGACAGGCGTAGCC
28S F	CTGTCCCTACCTACTATCCA
28S R	CTCCCACTTATTCTACACCT
18S F	CGGCTACCACATCCAAGGAA
18S R	GCTGGAATTACCGCGGCT
ND1-F	CTAGCAGAAACAAACCGGGC
ND1-R	CCGGCTGCGTATTCTACGTT
β-globin-F	GAAGCGATTCTAGGGAGCAG
β-globin-R	GGAGCAGCGATTCTGAGTAGA

F: forward, R: reverse.

Table S2. siRNA sequences against specific genes used in this study.

Genes	Sequence (5'-3')
Mfn1 1s	CCUUGAUGCUGAUGUCUUUTT
Mfn1 1as	AAAGACAUCAGCAUCAAGGTT
Mfn1 2s	GCAGAAGGAUUUCAAGCAATT
Mfn1 2as	UUGCUUGAAAUCCUUCUGCTT
H3f3a 1s	CGUUCAUUUGUGUGUGAAUUUUU
H3f3a 1as	AAAUUCACACACAAAUGAACGUU
H3f3a 2s	GCGAGAAAUUGCUCAGGACUUUU
H3f3a 2as	AAGUCCUGAGCAAUUUCUCGCUU
H3f3b 1s	UCUGAGAGAGAUCCGUCGUUAUU
H3f3b 1as	UAACGACGGAUCUCUCUCAGAUU
H3f3b 2s	GAAGCUGCCAUUCCAGAGAUUUU
H3f3b 2as	AAUCUCUGGAAUGGCAGCUUCUU
Neg s	UUC UCC GAA CGU GUC ACG UUU
Neg as	ACG UGA CAC GUU CGG AGA AUU

H3.3: H3.3A (*H3f3a*), H3.3B (*H3f3b*); *Neg*: negative control siRNA sequences

Table S3. Information on antibodies used in this study.

Antibodies	Vendor	Catalog number
Anti-MFN1 antibody	Proteintech	13798-1-AP
Anti-H3.3 antibody	Active motif	91191
Anti-H3.3S31p antibody	Active Motif	39637
Anti-RNA Pol II antibody	Active Motif	39097
Anti-Ser2p antibody	Abcam	ab193468
Anti-H4 antibody	ABclonal	A23000
Anti-PADI6 antibody	LSBio	LS-C695606
Anti-TLE6 antibody	Abmart	M034864
Anti-OOEP antibody [1]	N/A	N/A
Phalloidin-FITC (F-actin)	Sigma-Aldrich	P5282
Anti-RPL7 antibody	Proteintech	14583-1-AP
Anti-RPL3 antibody	Proteintech	11005-1-AP
Anti-H3K27me3 antibody	Diagenode	C15410069
Anti-H3K4me3 antibody	CST	9751S
Anti-H3K27ac antibody	Abcam	ab177178
Anti-H3K9ac antibody	Abcam	ab32129
Anti-H3K36me3 antibody	Abclonal	A2366
Anti-Lamin B1 antibody	Santa Cruz	sc-30264
Goat anti-mouse IgG (H+L)	Yeasen	33912ES60
Goat anti-rabbit IgG (H+L)	Yeasen	33112ES60
Anti-rabbit IgG for IP (HRP)	Vazyme	RA1008
Anti-mouse IgG for IP (HRP)	Vazyme	RA1009

Reference

1. Li L, Baibakov B, Dean J. A subcortical maternal complex essential for preimplantation mouse embryogenesis. *Dev Cell* 2008, **15**(3)**:** 416-425.
